# The Current Burden of Carbapenemases: Review of Significant Properties and Dissemination among Gram-Negative Bacteria

**DOI:** 10.3390/antibiotics9040186

**Published:** 2020-04-16

**Authors:** Dalal Hammoudi Halat, Carole Ayoub Moubareck

**Affiliations:** 1 Department of Pharmaceutical Sciences, School of Pharmacy, Lebanese International University, Bekaa Campuses, Beirut, Lebanon; dalal.hammoudi@liu.edu.lb; 2 College of Natural and Health Sciences, Zayed University, Dubai, UAE

**Keywords:** carbapenemases, β-lactamases, Gram-negative bacteria, resistance, KPC, metallo-β-lactamases, oxacillinases

## Abstract

Carbapenemases are β-lactamases belonging to different Ambler classes (A, B, D) and can be encoded by both chromosomal and plasmid-mediated genes. These enzymes represent the most potent β-lactamases, which hydrolyze a broad variety of β-lactams, including carbapenems, cephalosporins, penicillin, and aztreonam. The major issues associated with carbapenemase production are clinical due to compromising the activity of the last resort antibiotics used for treating serious infections, and epidemiological due to their dissemination into various bacteria across almost all geographic regions. Carbapenemase-producing *Enterobacteriaceae* have received more attention upon their first report in the early 1990s. Currently, there is increased awareness of the impact of nonfermenting bacteria, such as *Acinetobacter baumannii* and *Pseudomonas aeruginosa*, as well as other Gram-negative bacteria that are carbapenemase-producers. Outside the scope of clinical importance, carbapenemases are also detected in bacteria from environmental and zoonotic niches, which raises greater concerns over their prevalence, and the need for public health measures to control consequences of their propagation. The aims of the current review are to define and categorize the different families of carbapenemases, and to overview the main lines of their spread across different bacterial groups.

## 1. Introduction and Historical Perspective of Carbapenemases

Since the discovery of penicillin early in the previous century, Gram-negative bacteria have become proficient at evading the bactericidal activity of β-lactam antibiotics, principally through production of β-lactamases. Over the past decades, the emergence and dissemination of bacterial pathogens that are resistant to carbapenems, which are the broadest spectrum agents of the β-lactam group, has become apparent as a worldwide public health issue. The increasing prevalence of such organisms threatens to restrain treatment options after compromising carbapenems, which is often regarded as “last resort” antimicrobials in hospitals and long-term care facilities [1]. A primary mechanism of carbapenem resistance in Gram-negative bacteria is the production of acquired carbapenemases, which are β-lactamases with the widest spectrum of activity [2]. In addition to hydrolyzing carbapenems, these enzymes are active against most other members of the β-lactam group with few exceptions. The major drive behind the emergence of carbapenemases was the widespread use of carbapenems in treating serious infections caused by extended-spectrum β-lactamase (ESBL) producing-pathogens [3]. Such selection pressure on bacterial pathogens has rendered these pathogens carbapenem-resistant as a predictable consequence [4]. Carbapenemases are frequently found on mobile genetic elements and have the potential to be widespread all over the world, with the situation in many countries still not well documented [5]. Bacterial isolates harboring carbapenemases are often resistant to multiple antibiotic classes, and, with a narrow pipeline of novel agents in the near horizon, the need arises to better comprehend carbapenemases and to push for the development of containment strategies to reduce their spread [2].

Carbapenemases are the most resilient β-lactamases. Historically, carbapenemases were originally described in Gram-positive bacteria such as *Bacillus cereus* and *Bacillus licheniformis* [6,7], and, unlike other β-lactamases known at that time, they were inhibited by ethylenediaminetetraacetic acid (EDTA), which establishes them as metalloenzymes. Later work proved that all metallocarbapenemases contain at least one zinc atom at the active site, which facilitates hydrolysis of β-lactams [8]. Additionally, carbapenemases occasionally existed in *Strenotrophomonas maltophilia* and other clinical isolates were considered an infrequent cause of human infections [9]. However, during the early 1990s, intermittent reports started to describe carbapenemases among members of the family *Enterobacteriaceae*, with the initial description of a chromosomal imipenem-hydrolyzing enzyme in *Serratia marcescens* from the United Kingdom [10] and of another chromosomal NmcA (not metalloenzyme carbapenemase A) in *Enterobacter cloacae* from France [11,12]. Contrary to other sequenced carbapenemases, the latter was a class A serine β-lactamase. Plasmid-encoded resistance to carbapenems soon emerged in Japan [13], Greece [14], Portugal [15], and Italy [16] with reports of metallo-β-lactamases (MBLs) from *Pseudomonas aeruginosa*, replicated as well in some countries from Latin America [17]. An epidemic of multidrug resistant MBL-producing Gram-negative pathogens was then predicted [4]. In spite of the expansion of MBL families until the beginning of the twenty-first century, reports tended to describe mostly little outbreaks, restricted in both duration and the geographic region [18,19,20].

The more recent wave of spreading of carbapenemases has been ongoing for the preceding 20 years. First, in 2001, with identification of KPC (*Klebsiella pneumoniae* carbapenemase) initially in the United States in *K. pneumonia* isolates [21,22], and its description in other areas in the world and across different genera of *Enterobacteriaceae* [4,23]. Infections caused by organisms producing KPCs have limited treatment options, and are associated with poor clinical outcomes and high morbidity and mortality, which complicates their dissemination. This is currently considered global [24]. Second, in 2004, a transferrable carbapenemase, oxacillinase (OXA)-48, was isolated in Turkey from *K. pneumoniae*, and it hydrolyzed imipenem and was remotely related to other oxacillinases [25]. The plasmid carrying this carbapenemase has been described to possess derepressed transfer properties [26], which allows for an efficient intercontinental spread among *Enterobacteriaceae* [27]. Three other oxacillinase gene clusters have been described in *Acinetobacter baumannii*, which correspond to *bla*_OXA-23_-like, *bla*_OXA-40_-like, and *bla*_OXA-58_-like genes [28]. The *bla*_OXA-23_ gene, which was first characterized in 1995 in Scotland and initially named ARI-1 (*Acinetobacter* resistant to imipenem) [29], was a serine β-lactamase [30] and has been increasingly reported worldwide. Third, and apart from KPC and OXA enzymes, the year 2009 witnessed the significant description of NDM (New Delhi metallo-β-lactamase) from a Swedish patient hospitalized in India [31]. This preliminary emergence of NDM-1 has now escalated to be conveyed in all continents, often in patients with history of travel or hospitalization in the Indian subcontinent [32].

So far, the most effective carbapenemases, in terms of carbapenem hydrolysis and geographical spread, are KPC, OXA-48, and the MBLs VIM, IMP, and NDM [33]. Additional carbapenemases and new members from the different families are yet to be discovered. Projections into the future are that Gram-negative pathogens will continue to accumulate multiple carbapenemase-encoding genes [34]. Clinically, this will be reflected as increased carbapenem inhibitory concentrations, ruling out the available therapeutic choice against such multi-armored pathogens, which is the combined treatment including at least one carbapenem [35]. Accordingly, there is an urgent need to continuously update the current knowledge regarding carbapenemases as major features of β-lactam resistance. This review highlights the current understanding of carbapenemases from microbiological and epidemiologic viewpoints, with emphasis on their molecular and genetic properties, as well as their species and geographical distribution.

## 2. Classification

Like other β-lactamases, carbapenemases can be classified according to two possible schemes: functional and molecular. The functional scheme, known as the Bush-Jacoby-Medeiros classification, is a biochemical scheme based on properties like isoelectric points, substrate profiles and inhibitor characteristics [36]. Accordingly, β-lactamases are functionally classified into groups 1–4 with many subgroups under group 2. This classification has continuously matured over the years [37] and was updated in 2010 in which several new subgroups of each of the major groups were described [38]. According to this last update, group 1 includes cephalosporinases, which are more active on cephalosporins than benzylpenicillin and are usually resistant to inhibition by clavulanic acid. They also have high affinity to aztreonam [39]. Group 2 includes the largest group of β-lactamases. In this group, subgroup 2a includes penicillinases predominantly present in Gram-positive cocci [38], while subgroup 2b includes β-lactamases that readily hydrolyze penicillin and early cephalosporins, and are strongly inhibited by clavulanic acid and tazobactam. Subgroup 2c encompasses penicillinases that are characterized functionally by the ability to hydrolyze carbenicillin or ticarcillin more than benzylpenicillin, and by being easily inhibited by clavulanic acid or tazobactam [36]. Subgroup 2d includes enzymes able to hydrolyze cloxacillin or oxacillin at a rate of 50% greater than that for benzylpenicillin and, hence, are known as OXA enzymes [38]. Subgroup 2e includes cephalosporinases that have the ability to hydrolyze extended-spectrum cephalosporins and are inhibited by clavulanic acid or tazobactam. They can be differentiated from group 1 enzymes by their poor affinity for aztreonam [40]. Subgroup 2f includes serine carbapenemases that can be inhibited by tazobactam better than by clavulanic acid [8], while group 3 includes metallo-β-lactamases distinguished by their zinc ion requirement at the active site. The last updated classification scheme did omit group 4 β-lactamases previously included in the functional classification, as they most likely would be included in one of the enzyme groups 1 to 3, until more information about them becomes available [38]. As for carbapenemases, these mainly fall under functional groups 2d, 2f, or 3 [1].

According to the more commonly used Ambler classification system, β-lactamases are categorized into molecular classes A, C, and D, which include the β-lactamases with serine at their active site, whereas molecular class B are all metalloenzymes with a zinc active site. This structural classification is based on amino acid homology but lacks detail concerning enzymatic activity [8]. In this system, carbapenemases are categorized as classes A, B, and D. Rare carbapenemases belonging to Ambler class C exist, as described below. These usually cause reduced susceptibility to carbapenems as a consequence of the low enzyme’s catalytic efficiency and a permeability defect in the bacterial cell [35]. The description below shows molecular properties, inhibitors, genetic background, and major examples of each of the Ambler classes of carbapenemases. Examples of major carbapenemase groups with data on their original discovery and genetic location are listed in Table 1.

## 3. Ambler Class A Carbapenemases

A number of carbapenemases belonging to Ambler class A have been identified with broad hydrolytic profile for all β-lactams except cephamycins. As such, they mediate serine-directed hydrolysis of aminopenicillins, ureidopenicillins, first-generation and second-generation cephalosporins, aztreonam, and carbapenems [41]. Bacteria expressing these enzymes are characterized by reduced susceptibility to imipenem, with minimum inhibitory concentration (MICs) ranging from mildly elevated to fully resistant. These carbapenemases, therefore, may go unrecognized following routine susceptibility testing [8]. They are inhibited by clavulanate, tazobactam and boronic acid derivatives [41].

Some Ambler class A carbapenemases are chromosomally encoded like NmcA [11], SME-1 (*Serratia marcescens* enzyme-1), first described in London [42], in addition to less commonly described enzymes like SFC-1 (*Serratia fonticola* carbapenemase-1) [43], SHV-38 (sulfydryl variable-38) [44], and PenA [45]. SFC-1 has been described in an environmental isolate in Portugal, while SHV-38 was isolated from a clinical *K. pneumoniae* isolate in Paris, and had an alanine to valine substitution in position 146 compared to the ESBL SHV-1, which forms an infrequent example of SHV-type β-lactamase capable of hydrolyzing imipenem. PenA is a worrisome type of carbapenemase described in the *Burkholderia cepacia* complex, and can peculiarly hydrolyze inhibitors and all β-lactam antibiotics. Very recently in 2019, the variant SME-4 was encoded by the chromosome of *S. marcescens* from Argentina [46]. Such chromosomally encoded class A carbapenemases have never been described on transferable genetic elements, which likely justifies their sporadic reports worldwide [45].

Other class A carbapenemases are plasmid-encoded like IMI (imipenemase), KPC, and some varieties of GES (Guiana extended spectrum). The plasmid-encoded enzymes are often associated with mobile elements responsible for their genetic transfer [8]. The first IMI-1 was identified in an *E. cloacae* from California, and was chromosomally encoded [47]. In contrast, IMI-2 and IMI-3 have been described as plasmid-mediated enzymes in *Enterobacter asburiae* and *E. cloacae*, respectively [45]. More recently, defined variants of this carbapenemase encoded by *bla*_IMI-5_ and *bla*_IMI-6_, were reported in a Canadian study [48].

Perhaps the most troublesome carbapenemase of Ambler class A is KPC because of its location on self-conjugative plasmids, and its frequent association with *K. pneumoniae*, an organism notorious for its ability to accumulate and transfer resistance determinants [1,45]. The first enzyme, KPC-1, was isolated from a *K. pneumoniae* isolate in North Carolina in 1996 [21]. It showed 45% identity to SME-1, and hydrolyzed not only carbapenems but also penicillins, cephalosporins, and monobactams, while showing the highest affinity for meropenem. Soon thereafter, outbreaks of *K. pneumoniae* harboring KPC-2 [49] and KPC-3 [49,50] were described from New York city. Subsequent revision of *bla*_KPC-1_ sequence demonstrated that KPC-1 and KPC-2 are identical enzymes, while KPC-3 differs from KPC-2 (formerly KPC-1) by one amino acid change of histidine to tyrosine [50]. Both KPC-2 and KPC-3 continued to be isolated in the Eastern parts of the US and disseminated across other *Enterobacteriaceae* genera as a result of clonal expansion and horizontal gene transfer [51]. Within a few years, KPC producers became global, with reports from North and South Americas, the Middle East, Greece, Italy, and China where they are now considered endemic [52]. Although, so far, more than 20 different KPC variants have been described, KPC-2 and KPC-3 persist as the most common. In *K. pneumoniae*, the worldwide spread of the *bla*_KPC_ genes is currently linked to a major clone (sequence type ST-258), which serves as a successful transporter [45]. However, within a given geographical location, several other KPC clones may disseminate, differing by Multi Locus Sequence Typing (MLST) type, such as ST512 in Finland [53], ST307 in Puerto Rico [54], ST11 in Singapore [55], and others. Despite such genetic diversity, the *bla*_KPC_ genes are generally associated to a single transposon, Tn*4401*. This is a 10-kb Tn3-based transposon, delimited by two 39-bp imperfect inverted repeat sequences, that harbors, in addition to the KPC-2 gene, a transposase gene, a resolvase gene, and two novel insertion sequences known as IS*Kpn6* and IS*Kpn7* [56]. Besides *Enterobacteriaceae*, KPC has been described in *P. aeruginosa* from Brazil [57] and China [58], as well as in *A. baumannii* from Puerto Rico [59] and Portugal [60]. KPC-producing *K. pneumoniae* isolates may be deficient in OmpK35 and OmpK36, which are porins for carbapenem entry. This further amplifies resistance to carbapenems [61,62]. On another note, KPC-producing *E. coli* have been reported in wastewater treatment plants in China [63] as well as in hospital wastewater and riverbeds in South Africa [64]. Examples of KPC-producers as well as other carbapenemase-producing Gram-negative bacteria from environmental and animal samples are shown in Table 2.

The GES family of enzymes are encoded by genes on integrons or plasmids, and were originally classified as ESBLs. Eventually, the substrate profile of some members of this family was expanded to include carbapenems, after isolation of GES-2 producing nosocomial *P. aeruginosa* with limited susceptibility to imipenem in 2001 [65]. The probable change in the substrate profile was attributed to a single amino acid substitution of the original enzyme, GES-1. Later, over 20 GES variants were described, but only a few display carbapenemase activity. For instance, *P. aeruginosa* from Spain [66] and Dubai [67] revealed GES-5, while *P. aeruginosa* from Mexico revealed GES-20 [68]. In addition, Moubareck and Colleagues identified GES-11 in an isolate of *A. baumannii* in France [69], and the same enzyme was identified in nosocomial isolates of this organism from Lebanon [70]. In spite of the fact that they are plasmid mediated, GES carbapenemases do not have aptitude for efficacious dissemination like KPC [71].

## 4. Ambler Class B Carbapenemases (Metallo-β-Lactamases)

Unlike the serine-dependent carbapenemases belonging to classes A and D, class B carbapenemases are MBLs that require a heavy metal like zinc for catalysis. MBLs have a broad substrate spectrum and can catalyze hydrolysis of virtually all β-lactam antibiotics including carbapenems with the exception of monobactams, such as aztreonam [72]. Sophisticated modeling analyses show that the active site of these enzymes is defined by a peculiar set of amino acids and contains one or more bounded zinc ions that are central to the catalytic mechanism. These ions usually coordinate two water molecules necessary for hydrolysis, and the active site orients and polarizes the β-lactam bond to facilitate nucleophilic attack by zinc-bound water/hydroxides. The wide, plastic, active-site groove can accommodate most β-lactam substrates, which explains the broad spectrum of activity [73]. Because MBLs are metalloenzymes, they are resistant to the commercially available β-lactamase inhibitors but are susceptible to inhibition by metal ion chelators like ethylenediaminetetraacetic acid (EDTA) [8].

Originally, the first MBLs were ubiquitous chromosomal enzymes identified more than 50 years ago in environmental and opportunistic pathogenic bacteria such as *B. cereus*, *Aeromonas* spp., *Legionella gormanii*, *Pseudomonas stutzeri*, *Shewanella* spp., and *S. maltophilia* [8,73,74]. With the exception of *S. maltophilia*, these bacteria have not been frequently associated with serious nosocomial infections, which are generally opportunistic pathogens. The chromosomal metalloenzymes are not easily transferrable and have not extensively contributed to an epidemiological burden of MBLs. Perhaps one exception to such chromosomal MBLs that represents a transferrable group is that from *Bacteroides fragilis*. This anaerobe is relatively resistant to β-lactams, and its MBL. Designated CfiA was first genetically characterized in 1990 and is one of the most intensely studied with respect to a mechanism of catalysis and structure-function properties [75]. Insertion elements, such as IS*942*, IS*1186*, and IS*4351*, have been identified immediately upstream of the ribosome-binding site of the gene encoding CfiA, which provides enhanced transcriptional capabilities for the gene [76,77]. Currently, the most common MBL families include the IMP, VIM, NDM, GIM, and SIM enzymes, which are genetically located within a variety of integrons, where they have been incorporated as gene cassettes. When these integrons become associated with plasmids or transposons, transfer between bacteria is readily facilitated [78]. MBLs are currently found in different Gram-negative bacterial species, and their presence is often associated with resistance to antibiotic classes, which results in multidrug resistance and comprises treatment options [79].

The first transferrable imipenem resistance through MBLs was reported from *P. aeruginosa* in 1991 in Japan [13], was designated IMP-1, and its gene was located on a conjugative plasmid conferring resistance to β-lactams, gentamicin, and sulfonamide. This plasmid was transferable by conjugation to *P. aeruginosa* but not to *Escherichia coli*. Soon, the same enzyme was identified in *S. marcescens* in Japan as well, and the area of Southeastern Asia remained, until today, the greatest reservoir of IMP-type enzymes [73]. Several years later, the same enzyme appeared in Italy in *A. baumannii*, representing its first description from European countries [80]. Additionally, IMP-2 from Italy and IMP-5 from Portugal were soon described [81] as well as IMP-4 from USA [82], both IMP-1 and 2 from the Middle East [83], and IMP-7 from Australia [84], which indicates that IMP genes were not solely restricted to the Far East region. Currently, the number of IMP varieties have reached about 50. Analysis of the genetic platform of *bla*_IMP_ genes has shown that most of them are embedded as gene cassettes in class I integrons, which harbors other resistance genes, such as *aac* (mediating resistance to aminoglycosides), *bla*_OXA_ (different serine oxacillinases, described shortly), and genes conferring resistance to antiseptics, or to chloramphenicol [8,78]. Apart from detection of *bla*_IMP_ in clinical isolates, zoonotic IMP-4 was described in *Salmonella enterica* serovar Typhimurium from companion cats in Australia [85]. Likewise, IMP-27 was isolated from environmental and fecal samples of *Enterobacteriaceae* recovered from swine operation farms in the US [86]. Therefore, IMP carbapenemases may play a role in a dissemination cycle that encompasses humans, animals, and the environment.

The second dominant group of MBLs are the VIM (Verona intergon-encoded MBL) category, so named due to initial discovery of VIM-1 in a *P. aeruginosa* isolate from Verona, Italy, in 1997 [16]. Similar to *bla*_IMP_, *bla*_VIM_ was also carried on a gene cassette inserted into a class 1 integron, and, when cloned into *E. coli*, resulted in a significant decrease in susceptibility to a broad array of beta-lactams (ampicillin, carbenicillin, piperacillin, mezlocillin, cefotaxime, cefoxitin, ceftazidime, cefoperazone, cefepime, and carbapenems) [16]. Soon afterward, VIM-2 was isolated in Marseille, France [87] from *P. aeruginosa* and shared 90% similarity with VIM-1. Although VIM-1 and VIM-2 have been identified in enterobacterial species. *P. aeruginosa* remains the most important known reservoir of these enzymes [73]. There are more than 40 allelic variants of VIM enzymes reported so far, and they mainly belong to three phylogenetic clusters: VIM-1-like, VIM-2-like, and VIM-7-like enzymes [88]. VIM-2-like enzymes have been associated mostly with *P. aeruginosa*, whereas VIM-1-like enzymes, in particular, VIM-4, have been reported in *Enterobacteriaceae.* The latest VIM-type to be fully characterized is VIM-7, which has been characterized from a carbapenem-resistant *P. aeruginosa* isolate from Houston, Texas [89]. It shares only a 77% identity with VIM-1 and 74% with VIM-2, and lies on a 24-kb plasmid, which can be readily transferred into *Enterobacteriaceae* and other pseudomonads, and was thought to originate from a different ancestral source. After original detection in North America, VIM-7 was recently described in another report from Brazil [90]. Our group has lately detected several VIM enzymes including VIM-2, VIM-30, VIM-31, and VIM-42 from nosocomial *P. aeruginosa* in Dubai, United Arab Emirates [67], with VIM-2 co-existing with the Ambler class A carbapenemase GES-5. Moreover, in Lebanon, we detected VIM-2 in 16% of a nationwide collection of nosocomial *P. aeruginosa* that were primarily spread among different hospitals by clonal dissemination [83]. The *bla*_VIM_ genes are typically embedded in class 1 integrons, which can be incorporated in either plasmids or chromosomes. Plasmids carrying *bla*_VIM_ genes in *Enterobacterriaceae* belong, most frequently, to the IncA/C or IncN group [91].

The spectacular global spread of NDM remains one of the most worrisome antibiotic resistance events caused by MBLs. The initial enzyme, NDM-1, was originally isolated from a carbapenem-resistant *K. pneumoniae* recovered from urine of a Swedish patient who had recently travelled to New Delhi [31]. NDM-1 was transferrable and shared little identity with other MBLs. The most similar MBLs were VIM-1/VIM-2 with which it has only 32.4% similarity, and can hydrolyze all β-lactams except aztreonam. Compared to VIM-2, NDM-1 displays tighter binding to most cephalosporins, such as cefuroxime, cefotaxime, and cephalothin (cefalotin), and also to penicillin [92]. By mid-2010, the NDM-1 gene may have been acquired by bacteria from the Indian subcontinent and introduced to other countries including European countries and the United States through tourists travelling around the globe. Yet, the Indian subcontinent remains as the principle reservoir [93]. A second probable epicenter seems to be located in the central Balkans without a clear connection to that of India [35]. NDM-genes are dominant in *K. pneumoniae* and *E. coli* isolates with certain sequence types (for *K. pneumoniae*, ST11, ST14, ST15, or ST147; for *E. coli*, ST167, ST410, or ST617) being the most prevalent [94]. However, variants of NDM have also been found in association with *A. baumannii* and *P. aeruginosa* [95]. NDM has been detected on surveillance specimens from hospital equipment [96], in environmental samples [97], in poultry farms [98], and from injured war victims in the Middle East [99].

As of 2020, 24 NDM variants have been identified in >60 species of 11 bacterial families, and several variants have enhanced carbapenemase activity with most *bla*_NDM_-carrying plasmids belonging to limited replicon types (IncX3, IncFII, or IncC) [94]. Examples of such variants are NDM-2 [100], NDM-3 [101], NDM-4 [102], and NDM-5 [103]. While commonly used phenotypic tests cannot reliably identify NDM, immunoassays can specifically detect these enzymes [104,105], and molecular approaches remain as the reference methods [106]. NDM will remain a severe challenge in health care settings, and more studies on appropriate countermeasures are required.

In 2002, Toleman and Colleagues described a novel MBL from a *P. aeruginosa* isolate in Sao Paulo, Brazil, distinctly different from VIM and IMP and, accordingly, represents a new subfamily. The enzyme was termed SPM-1 (Sao Paulo MBL-1) [107]. The SPM-1 gene was detected on a transmissible plasmid, and the enzyme contains the classic MBL zinc-binding motif and shows the highest identity (35.5%) to IMP-1. SPM-1 has been repeatedly detected in South America from *P. aeruginosa* [108,109,110], and has bypassed nosocomial prevalence to animals where it was recently isolated from *P. aeruginosa* existing as a part of normal microbiota of birds [111]. Sporadic reports of SPM-1 exist for *P. aeruginosa* from UK [112] and Switzerland [113] and *A. baumannii* from Iran [114]. Although some expert opinions have suggested the probability of wider spread by SPM [115], this carbapenemase is still mainly prominent in South American hospital outbreaks. The *bla*_SPM_ gene can be either chromosomal or plasmid-encoded, and is associated with the insertion sequence IS*CR4* (for common region 4) [116]. These elements are usually harbored in transposons and/or plasmids, which forms mobile vesicles for horizontal transfer of captured MBL genes between bacteria [117].

Other MBL-encoding genes include GIM (German imipenemase) described in Dusseldorf, Germany, from *P. aeruginosa* [118] on a 22-kb nontransferable plasmid. Unlike other class B carbapenemases, GIM has two zinc ions in the active site, but appears to be a weaker enzyme [73]. GIM has been lately identified in *A. baumannii* from Indian patients with severe urinary tract infection, sometimes concurrently with VIM [119]. Another MBL, SIM-1 (Seoul imipenemase-1) was isolated in Korea [120] and was carried on a gene cassette inserted into a class 1 integron, found in *A. baumannii* strains isolated in sputum and the urine of patients with pneumonia and urinary tract infections, respectively. In 2019, the spread of *bla*_SIM-1_ to *Enterobacteriaceae* has been suggested after a report from China indicating a mega-plasmid harboring this gene from a clinical *K. pneumoniae* isolate [121]. More recently, described MBLs include DIM-1 (Dutch imipenemase-1) [122], KHM-1 (Kyorin University Hospital MBL-1) [123], and TMB-1 (Triploi MBL-1) [124] that have been reported on occasional encounters. Future investigations are needed to unravel the epidemiology and propagation potential of such MBLs.

## 5. Ambler Class D Carbapenemases (Oxacillinases)

Among the earliest β-lactamases detected, class D β-lactamases were relatively rare and always plasmid mediated [125]. They were also referred to as oxacillinases because they commonly hydrolyze isoxazolylpenicillins such as oxacillin, methicillin, and cloxacillin much faster than classical penicillin, such as benzylpenicillin, and are relatively less effective against first-generation cephalosporins. The designation, OXA, thus, refers to the preferred substrate, which is oxacillin [126]. The active site of these enzymes includes a highly conserved serine-based structure, even though the rest of the molecule shows variability in the amino acid sequences, and they are characteristically not inhibited by β-lactamase inhibitors like clavulanate, sulbactam, tazobactam, cloxacillin, or metal chelators like EDTA [1]. The emergence of such enzymes presumably coincided with the widespread introduction of flucloxacillin and methicillin for treating staphylococcal infections. The early OXA β-lactamases such as OXA-1, OXA-2, and OXA-3 were plasmid-encoded and identified in Gram-negative bacteria. They were essentially penicillanases but hydrolyzed oxacillin is better than penicillin [127]. Later, OXA-11, which is the first extended-spectrum OXA variant, was isolated from *P. aeruginosa* [128], and exhibited a transferrable resistance profile with enhanced ceftazidime hydrolysis. Soon, other extended-spectrum OXA enzymes appeared like OXA-13, OXA-14, OXA-15, OXA-16, OXA-17, OXA-19, OXA-28, and OXA-45, which all remained confined to *P. aeruginosa*, and did not seem to be spreading [8,125]. Currently, OXA enzymes with carbapenem-hydrolyzing activity mainly include, among others, the groups OXA-23-like, OXA-24/40-like, OXA-48-like, OXA-58-like, OXA-143-like, and OXA-235.

The first carbapenem-resistant OXA-type β-lactamase, OXA-23, was identified on a large plasmid of a multidrug resistant (MDR)-*A. baumannii* collected from the blood of a patient hospitalized at the Edinburgh Royal Infirmary, Scotland, in 1985 [29], which is the same year when imipenem was approved for clinical use. After the sequence for this enzyme was published in the year 2000 [30], several *bla*_OXA-23-like_ genes (*bla*_OXA-23_, *bla*_OXA-102_, *bla*_OXA-103_, *bla*_OXA-105_, *bla*_OXA-133_, and *bla*_OXA-134_) were discovered on the chromosome of *Acinetobacter radioresistens*. This is a commensal bacterial species that resides on the skin of hospitalized and healthy individuals, and indicates that this species may be the probable natural reservoir of these enzymes [129]. Hydrolytic activity measurements done on OXA-23-like enzymes including OXA-23, OXA-27, and OXA-146 show considerable kinetic variation. However, the enzymes are able to hydrolyze oxyiminocephalosporins, aminopenicillins, piperacillin, oxacillin, and aztreonam in addition to carbapenems [130]. Among the carbapenems, OXA-23 has a much higher turnover rate for imipenem than for meropenem, ertapenem, or doripenem [131]. Despite the relatively low turnover rates for carbapenems displayed by these enzymes, the production of OXA-23 by an *A. baumannii* strain is enough to increase the MIC breakpoint for considering strains to be resistant. However, when OXA-23 is produced in a strain that also expresses the AdeABC efflux pump, the MICs are significantly elevated. This indicates that, unlike some of the other OXA-type carbapenemases, strains do not require other resistance mechanisms to work in synergy with OXA-23 to be carbapenem resistant. However, high resistance levels are achieved only when there are other mechanisms present [125]. The *bla*_OXA-23_ genes are usually located on plasmids and are flanked by two copies of the insertion sequences IS*Aba1* in opposite directions on the transmissible transposons Tn*2006* and Tn*2008*. They can also exist in association with the transposon Tn*2007*, which lacks the second copy of IS*Aba1*, and is associated instead with one copy of IS*Aba4* [132]. The transposon Tn*2009* has been detected in isolates from China [133,134], while Tn*2006* is the most commonly observed worldwide. The insertion sequences are likely to act as strong promotors for expression of OXA-23, which enhances carbapenem resistance [135]. Besides the location on plasmids, evidence shows chromosomal insertions of *bla*_OXA-23_ associated with transposon-mediated transmission [136].

More than 10 years after the initial discovery of OXA-23, a newly isolated enzyme named OXA-24, which was chromosomally encoded, was described [137]. It increased carbapenem MIC by four-folds, hydrolyzed benzylpenicillin, and was inhibited by chloride ions. OXA-24 was subsequently named OXA-40, and additional members of the group, namely OXA-25, OXA-26, OXA-72, and OXA-160 were subsequently discovered [125]. While the OXA-24 group is documented in *A. baumannii* in reports from certain countries like Taiwan [138], Thailand [139], Bulgaria [140], and Lebanon [141], it was also detected in *P. aeruginosa* [142] in one French study. It was previously thought that *bla*_OXA-24-like_ genes are mainly located on chromosomes and no mobile elements are associated. This does not definitely represent the current status of these genes. Recently, plasmids carrying OXA-24-like enzymes were detected in cattle [143] and in a domestic grey parrot presented to a veterinary clinic in Luxembourg [144]. This shows that some lineages of *A. baumannii* have successfully acquired OXA-24/40-encoding plasmids that emerged and started to appear in reservoirs including livestock, companion animals, and the environment.

In 2005, a carbapenem-hydrolyzing oxacillinase, OXA-58, was isolated from *A. baumannii* in Toulouse, France. This enzyme had only 50% amino acid similarity to other oxacillinases. It hydrolyzed penicillins, oxacillin, and imipenem but not expanded-spectrum cephalosporins. Its gene was plasmid-carried, and was found to be located within a transposon and bracketed by two copies of IS*Aba3* [145]. OXA-58 was reported in studies from Iran [146], Greece [147], Argentina [148], and Lebanon [149] in *A. baumannii* isolates. Although no more than three variants of this enzyme have been detected so far, including OXA-96, OXA-97, and OXA-164 [125], certain new observations may alter the known consensus for this enzyme. For example, although OXA-58 represents a class D carbapenemase that is extremely rare in *Entereobacteriaceae*, it has been reported in isolates of *Proteus mirabilis* in Belgium [150], in Germany [151], and, most recently, in Poland [152]. Mobilized plasmids allowing horizontal spread of the *bla*_OXA-58_ gene are suggested to allow its transfer among *P. mirabilis*, and the occurrence of these plasmids might suggest hidden dissemination of unknown scale and future potential. A second observation about OXA-58 comes from China, where a recent report discovered *bla*_OXA-58_ and *bla*_NDM-1_ was carried simultaneously on the same plasmid in *Acinetobacter pitti*, which is an emerging opportunistic nosocomial MDR pathogen [153]. This indicates the need for further investigations in the context of genomic epidemiological characteristics of OXA-58 as well as its clinical and microbiological significance on carbapenem resistance in *Acinetobacter* species. A third observation is the detection of OXA-58 in *Acinetobacter towneri* and in 9 environmental genera of bacteria from hospital sewage [154] and coastal water [155], respectively.

In 2004, a novel carbapenemase gene was detected in a carbapenem-resistant *A. baumannii* strain in Brazil [156]. The gene encoded a carbapenem-hydrolyzing class D beta-lactamase known as OXA-143, with 88% amino acid sequence identity with OXA-40, 63% identity with OXA-23, and 52% identity with OXA-58. It hydrolyzed penicillins, oxacillin, meropenem, and imipenem but did not expand spectrum cephalosporins. The gene was plasmid encoded but associated with neither insertion sequences nor integron structures. However, it was bracketed by similar replicase-encoding genes at both ends, which suggests acquisition through a homologous recombination process. Further identified variants of the OXA-143 group include OXA-182, OXA-231, OXA-253, and OXA-255. OXA-143 is still detected in Brazil [157,158], and, although believed that this country is the major epicenter, OXA-143 was detected in 2017 for the first time in 14% of *A. baumannii* isolates from teaching hospitals in the central part of Iran [159]. The variant OXA-231 was also isolated from a nosocomial *A. baumannii* isolate recovered from urine of a female patient, at Londrina, Brazil. This variant had reduced catalytic efficiency against carbapenems and noticeably increased the specificity for oxacillin [160].

In 2013, Higgins et al [161] reported three novel OXA enzymes, which include OXA-235 and its amino acid variants OXA-236 and OXA-237 in *A. baumannii* isolates from the United States and Mexico. The expression of OXA-235 resulted in reduced carbapenem susceptibility, while cephalosporin MICs were unaffected. Genetic analysis revealed that genes encoding the novel OXA variants were bracketed between two IS*Aba1* insertion sequences. Recently, results from the Canadian Nosocomial Infection Surveillance Program reported OXA-235 to account for almost half of all carbapenemases detected among *Acinetobacter* species from Canadian hospitals [162]. In a remarkable jump toward natural reservoirs, the variant OXA-278 of OXA-235 was detected in 2019 in *Acinetobacter lwoffii* from municipal wastewater treatment plants in Singapore [163].

Apart from the previous OXA families mostly described in *Acinetobacter*, OXA-48-like enzymes are particularly associated with *Enterobacteriaceae*, and likely designate one of the most concerning developments in carbapenem resistance in the last decade and are still globally ascending. First recovered from *K. pneumoniae* in Turkey [25], the OXA-48 enzyme has low-level hydrolytic activity against carbapenems, with much greater activity against imipenem than against meropenem, and results in only modest increases in MICs of the carbapenems [125]. It has very weak activity against expanded spectrum cephalosporins, and does not significantly hydrolyze ceftazidime and cefepime, but, in combination with impermeability, can lead to high-level resistance to carbapenems. OXA-48 significantly hydrolyzes only penicillin and narrow-spectrum cephalosporins. However, enteric Gram-negative bacteria with the *bla*_OXA-48-like_ genes could co-harbor genes encoding ESBL (*bla*_CTX-M_, *bla*_SHV_, *bla*_TEM_) or AmpC enzymes, or both, which confers nonsusceptibility to aztreonam, extended-spectrum cephalosporins, and carbapenem agents [1]. OXA-48 currently is widespread, not only in *K. pneumoniae*, but also in other *Enterobacteriaceae*. Turkey is reported as having the highest epidemiologic level of OXA-48, and other important reservoirs are linked to India, Middle East, and North African countries. Additionally, OXA-48 producers have been documented sporadically in several European countries, including France, Germany, Netherlands, Italy, Belgium, UK, Ireland, Slovenia, Switzerland, and Spain [33]. An “intercontinental spread” in Europe has been described, with endemic situation in certain countries [27]. In fact, the detection of OXA-48-like producers is difficult since the level of acquired resistance to carbapenems may remain very low, keeping these strains underreported. One concern for controlling the spread of OXA-48-like producers is the absence of phenotypic tests that could contribute to their laboratory recognition, since, like other class D carbapenemases, they are not inhibited by metal ion chelators or clavulanate.

The major vehicle carrying the *bla*_OXA-48_ gene has been identified. Complete sequencing of plasmid pOXA-48a carrying the gene from *K. pneumoniae* showed that its backbone corresponded to that of a 62.3 kb IncL/M-type plasmid in which the gene had been integrated through acquisition of the Tn*1999* composite transposon harboring the upstream and downstream IS*1999* insertion sequence, which acts as a promoter for *bla*_OXA-48_ gene expression [164]. Hence, the current spread of OXA-48 producers is related to the spread of such a single plasmid with a high conjugation rate among different enterobacterial isolates [165]. Nevertheless, chromosomal integration of OXA-48 was documented in *E. coli* from UK [166] and Egypt [167]. Several variants of OXA-48 were identified. OXA-48, OXA-181, OXA-232, OXA-204, OXA-162, OXA-163, and OXA-244, in that order, are the most common enzymes among the group [168]. Four of these, OXA-162, OXA-163, OXA-181, and OXA-232, have had their kinetic properties measured, and while OXA-163 very poorly hydrolyzes carbapenems, OXA-181 and OXA-232 appear broadly similar to OXA-48 in their activity, with OXA-232 demonstrating better hydrolysis of penicillin [125]. The OXA-181 variant, which differs from OXA-48 by four amino acid substitutions, is prominent in India and has been associated with other carbapenemase genes, including *bla*_NDM-1_ and *bla*_VIM-5_ [169]. Recently, in Tunisia, two independent research groups showed that OXA-204 has emerged in clinical *E. cloacae* [170], and also in *Citrobacter freundii* from wastewater [171]. Moreover, OXA-48-like enzymes were implicated in carbapenem resistance in wild mammals and birds from Catalonia, Spain [172] and in companion animals from Germany [173], which indicates variability of niches where such enzymes are disseminating. The respective features of each OXA-48-like variant and their respective impacts in terms of carbapenem resistance need to be continuously elucidated. Given the rapid spread of *Enterobacteriaceae* producing OXA-48-like enzymes in different ecosystems, and the increasing number of reservoirs for such organisms, not only in hospitals but also in the community, among animals and in the environment, the medical community should remain alert to the threats posed by these organisms. Detection should be optimized to reduce their spread.

## 6. Emerging Ambler Class C Carbapenemases

The Ambler class C β-lactamases (AmpC cephalosporinases) confer resistance to penicillin, oxyiminocephalosporins, cephamycins (cefoxitin and cefotetan), and, variably, to aztreonam [72]. An exceptional member with different properties is ACC-1, a plasmid-encoded class C β-lactamase identified in clinical isolates of *K. pneumoniae*, *P. mirabilis*, *Salmonella enterica*, and *E. coli*. Due to conformational alterations in this enzyme structure, ACC-1-producing bacteria are susceptible to cefoxitin, whereas they are resistant to oxyiminocephalosporins [174]. AmpC cephalosporinases are not significantly inhibited by other β-lactamase inhibitors such as clavulanic acid, but may be inhibited by boronic acid and cloxacillin [41]. They may be encoded by the chromosome of many *Enterobacteriaceae* like *E. cloacae* and *S. marcescens*, and a few non-enterobacterial organisms like *P. aeruginosa*. In such instances, they are inducible by antibiotics or expressed at high levels by mutation. Nevertheless, AmpC enzymes may also be acquired on transmissible plasmids, which, consequently, makes them appear in bacteria by lacking or poorly expressing a chromosomal *bla*_AmpC_ gene, such as *E. coli*, *K. pneumoniae*, and *P. mirabilis* [39].

Studies describe only a few AmpC cepahlosporinases with carbapenemase activity. For example, plasmidic CMY-2-type, ACT-1-type, and DHA-1-type AmpC enzymes may promote the emergence of carbapenem resistance in porin-deficient clinical isolates of *Enterobacteriaceae* [175,176]. Very recently, Jousset et al. showed that *E. cloacae* can chromosomally encode an AmpC enzyme of the type ACT-28. Kinetic parameters of purified ACT-28 revealed a slightly increased imipenem hydrolysis compared to that of ACT-1 [177]. CMY-10 was the first reported carbapenemase among plasmidic class C β-lactamases, and this enzyme was also a class C ESBL with extended substrate specificity for extended-spectrum cephalosporins [178]. In 2014, ADC-68 was reported in *A. baumannii* from Korea as a chromosomal class C β-lactamases that possesses class C extended-spectrum β-lactamase and carbapenemase activities [179]. Many reports showed that such rare AmpC with carbapenemase activity may aggravate carbapenem resistance when coupled with outer membrane permeability and/or efflux pump overproduction [70,176].

## 7. Distribution of Carbapenemases Among Gram-Negative Pathogens

**Figure 1:**
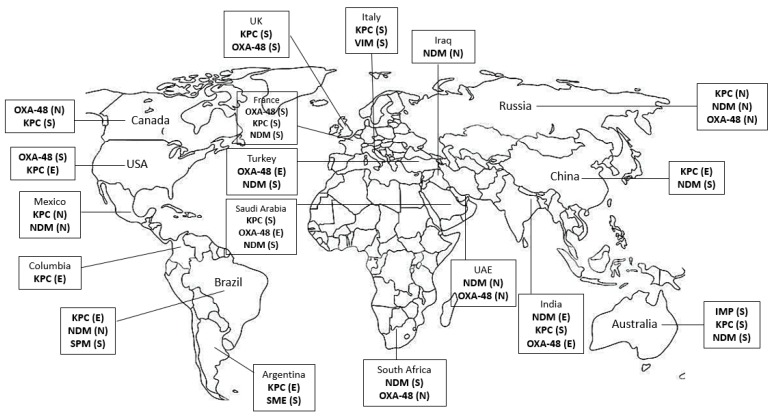
Worldwide distribution of KPC, NDM, VIM, IMP, SME, SPM, and OXA-48 carbapenemases with a status of dissemination. E = endemic. S = sporadic. N = newly detected. KPC = *Klebsiella pneumoniae* carbapenemase. NDM = New Delhi metallo-β-lactamase. VIM = Verona-intergon-encoded metallo-β-lactamase. IMP = imipenemase, SME = *Serratia marcescens* enzyme, SPM = Sao Paulo metallo-β-lactamase. OXA = oxacillinase.

The section below describes the carbapenemase groups in Gram-negative bacteria including *Enterobacteriaceae*, *Pseudomonas*, *Acinetobacter*, and others. A world map showing geographical distribution and endemicity of major carbapenemases is shown in Figure 1.

### 7.1. Enterobacteriaceae

Members of the family *Enterobacteriaceae*, such as *E. coli*, are normal inhabitants of the intestinal tract in humans and animals, and are commonly isolated in clinical cultures. They are the causative agent of several types of infections in humans, including respiratory tract infections, urinary tract infections, and bloodstream infections in hospitalized or otherwise immunocompromised subjects. In the context of antimicrobial resistance, *Enterobacteriaceae* are especially important as they are a cause of community-associated as well as healthcare-associated infections, which generates a major clinical and public health challenge [180]. The overdependence on carbapenems as empiric treatment in the management of infections caused by *Enterobacteriaeceae* was driven by cephalosporin resistance in this family due to production of ESBLs [181]. Carbapenems proved especially useful since ESBL producers are also resistant to other antibiotic classes including aminoglycosides, tetracyclines, and fluoroquinolones [71]. Unfortunately, this has led to the emergence of carbapenem-resistant *Enterobacteriaceae* (CRE), defined as bacteria belonging to the *Enterobacteriaceae* family that have the ability to survive and grow in the presence of clinically relevant concentrations of carbapenems [182]. From a therapeutic perspective, CRE represent a threat as only a few antibiotics retain activity against them. This is due to the ability of carbapenemases to hydrolyze most other β-lactam antibiotics, and to frequent coexistence in CRE isolates of additional mechanisms of resistance against other antibiotics such as fluoroquinolones and aminoglycosides [183]. To date, carbapenems are still considered a last-line of therapy against CRE, and, although few additional options exist, concerns over their efficacy and toxicity profiles are reported. In addition, rates of resistance to these agents such as tigecycline and polymyxins are increasing [184,185]. Currently, combination therapeutic strategies for CRE infections, include high-dose tigecycline, high-dose prolonged-infusion of carbapenem, and double carbapenem therapy. Newly available combinations like ceftazidime/avibactam are active against KPC and OXA-48 producers while meropenem/vaborbactam is active against KPC-producers. Plazomicin, which is a next-generation aminoglycoside, and eravacycline, which is a tetracycline class antibacterial, have in vitro activity against CRE [186].

*Enterobacteriaceae* can become resistant to carbapenems by three possible mechanisms: efflux pump overactivity, porin loss or mutation, and carbapenemase production, which remains the main resistance mechanism [187]. In fact, while carbapenemases specifically target carbapenems and other ß-lactam antibiotics, efflux pump expression or porin changes are associated with multi-drug resistance, and aim to block penetration of antibiotics into the bacterial cell [188]. Regarding efflux pumps, the resistance-nodulation-division (RND) group is a major mechanism of multi-drug resistance in *Enterobacteriaceae*. Among the different efflux systems, the AcrAB-TolC RND system is the most common, and has been identified among carbapenem-resistant *E. cloacae* [189], *E. coli* [190], and *K. pneumoniae* [191]. Alteration of porin synthesis, such as deficiency of OmpK-35 and 36 in *K. pneumoniae* [192,193], and loss of OmpF and OmpC in *E. coli* [194], have been described in AmpC- and carbapenemase-producing *Enterobacteriaceae*. Studies suggest that strains with porins mutated or downregulated typically do not have the potential for mobilization into community settings but may carry the risk of possible local proliferation within hospitals [188]. It is worth noting that CRE shows efflux activity or permeability lesions may express these mechanisms paired to production of other β-lactamases such as AmpC enzymes or ESBLs [181,193].

Nevertheless, carbapenemases are currently considered the major mechanism of resistance in CRE, even though this apparently was not the case around mid-2000s, when most carbapenemases were confined to *P. aeruginosa*, with only anecdotal reports in *Enterobacteriaceae* [73,195]. Afterward, and after the detection of the first strain of CRE in the 1990 [10], CRE had rapidly spread with different carbapenemases predominating in various geographic areas. For instance, NDM-1 is currently the main carbapenemase in India, Pakistan, and Sri Lanka, as well as in specific European countries, including Romania, Denmark, and Poland, mostly in *K. pneumoniae* and *E. coli* [71]. *Morganella morgani* was shown to harbor NDM-5 recently in reports from China [196], and GES-5 in reports from Brazil [197]. KPC-producing *Enterobacteriaceae* are endemic in the United States, Colombia, Argentina, Greece, and Italy, and cause epidemics in China and the Middle East [188]. On the other hand, OXA-48-like enzyme-producers are widespread in Turkey and surrounding countries as well as in the Middle-East and North Africa [33]. OXA-48-like producing *K. pneumoniae* clones have persisted in Turkey as a cause of nosocomial infections, and Turkey, according to the European Center for Disease Control and Prevention, which was reported as having the highest epidemiologic level (stage 5 “endemic situation”) of these strains in 2014–2015 [198]. Today, the extensive international movement and exchange has helped OXA-48 producing *Enterobacteriaceae* to spread from many Middle-Eastern countries into other parts of the world.

Among CRE, and from an epidemiological implication, the international spread of KPC- producing *K. pneumoniae* of a single multi-locus sequence type (ST), ST258, is alarming. This clone is responsible for the rapid increase in antimicrobial resistance among *K. pneumoniae* strains [199]. *K. pneumoniae* is a major cause of hospital-acquired infections including pneumonia, bloodstream infections, urinary tract infections, and infections in newborns and the intensive care unit. In some countries, because of resistance, carbapenem antibiotics are not efficient in more than half of the patients treated for *K. pneumoniae* infections [200], and therapy choices are limited to colistin, polymyxin B, fosfomycin, tigecycline, and selected aminoglycosides [23]. The worldwide spread of *K. pneumoniae* ST258 represents a prototype of the role of epidemic plasmids in the dissemination of pathogens set for global nosocomial dominance, This is similar to the successful international spread of *E.coli* ST131 associated with ESBL production, especially CTX-M-15 [201]. The *K. pneumoniae* ST258 is described as a “high-risk clone.” These clones are defined to show an enhanced ability to colonize, spread, and persist in a variety of niches. They have acquired certain adaptive traits that increase their pathogenicity and survival skills, and, at the expense of such adaptation, these strains have acquired antibiotic resistance pathways. They have the persistence and flexibility to accumulate and exchange resistance and virulence genes with other bacteria. High-risk clones have contributed to the propagation of different plasmids, genetic platforms, and resistance genes among Gram-negative bacteria [202,203]. In such a context, the *K. pneumoniae* ST258 pandemic has been distressing to the medical and scientific community. Originally, Kitchel and colleagues [204] showed in a nationwide study from the United States’ Centers for Disease Control and Prevention in 2009, that 70% of KPC-producing *K. pneumoniae* across all states belonged to a single dominant strain, ST258, which was identified by both pulsed-field gel electrophoresis (PFGE) and MLST. Soon thereafter, epidemiologic surveillance from different countries showed global spread of ST258 among *K. pneumoniae* isolates with KPC in numerous countries such as Mexico [205], Canada [206], Brazil [207], and Ecuador [208]. Outside the Americas, ST258 was detected in Spain [209], Greece [210], Germany [211], Italy [212], Norway [213], China [214], and Korea [215], which suggests that it possesses characteristics of an international high-risk clone. Apart from such a clinical phenomenon, a recent paper from Croatia [216] described the first evidence of KPC-2-producing *K. pneumoniae* of ST258 in river water, where it persisted for 50 days. This confirms the ability of environmental perseverance and dissemination.

The genetic support of *bla*_KPC_ in *K. pneumoniae* ST258 lies in a variety of plasmids, with the most commonly reported being an IncF plasmid with FII_K_ replicons, first named pKpQIL. This was a 113-kb IncF plasmid with an FII_K2_ replicon containing Tn*4401a*, and was isolated from a KPC-3-producing *K. pneumoniae* [217]. Later, pKpQIL-like plasmids from strains in the United States were described, and associated with *bla*_KPC-2_ and, to a lesser extent, with *bla*_KPC-3_. Additionally, findings demonstrated that pKpQIL plasmids are both spreading clonally in ST258 strains and transferred horizontally to different sequence types and species, which further highlights the clinical and public health concerns associated with this clone [218]. The ongoing *K. pneumoniae* ST258 pandemic is assumed to result from both plasmid-mediated spread that involves horizontal transmission of resistance genes between bacteria, as well as clonal expansion. Non-ST258 *K. pneumoniae* with *bla*_KPC_ did not demonstrate global success as ST258 with *bla*_KPC_. Therefore, global dissemination and survival of *K. pneumoniae* ST258 are partly dependent on the combination with *bla*_KPC_ on IncF plasmids with survival factors inherently present on the chromosome of this high-risk clone [200].

Overall, the worldwide genetic epidemiology of KPC-producing bacteria still shows that *K. pneumoniae* is the most common species and ST258 is the predominant clone. This suggests a unique fitness and selective advantage of this clone, which are far beyond simple antimicrobial resistance. It also suggests a capacity of this ST for high transmissibility, which likely requires immediate infection control actions and enhanced surveillance in favor of reducing the spread of KPC among *Enterobacteriaceae.*

### 7.2. Pseudomonas

A non-fermentative, and aerobic non-enteric Gram-negative bacterial pathogen, *P. aeruginosa* raises environmental, clinical, and global public health concerns due to its global presence, diverse ecological distribution, invasiveness, and life-threatening infections. It is widely distributed in the environment, and isolated from soil, organic matter, skin flora, water, plants, animal sources, moist surfaces, and medical equipment [219]. Infections with *P. aeruginosa* include pneumonia and sepsis, particularly in ventilated patients in intensive care units, and is still burdened with high morbidity and mortality. Additionally, it is associated with severe ocular, burn, and airway infections. It commonly infects patients with cystic fibrosis, chronic obstructive pulmonary disease (COPD), immunosuppressed organ transplant recipients, and/or those who underwent invasive medical procedures [220]. Intrinsically, *P. aeruginosa* is resistant to rifampin, tetracycline, chloramphenicol, trimethoprim-sulfamethoxazole, and many β-lactams. Low membrane permeability and efflux pump expression are partly responsible for such intrinsic resistance. As far as carbapenems are concerned, the Centers for Disease Control and Prevention reported in 2019 a carbapenem resistance rate of up to 12% in *P. aeruginosa* [221] while a PubMed search published in 2015 showed that international resistance rates of *P. aeruginosa* to carbapenems vary from 10% to 50% [222]. Although the rates varied from one country to another, the report estimates such strains to increase gradually due to both carbapenem use and medical applications, and the rate is estimated currently to be high enough to cause concern for public health microbiologists and infection specialists. Numerous resistance mechanisms drive carbapenem resistant phenotypes in Pseudomonas, most often including porin deficiency (especially OprD), efflux pump overactivity (mainly MexAB-OprM and MexCD-OprJ), and, less often, carbapenem-inactivating enzymes [223].

Regarding the latter mechanism, and in which lies the scope of this review, *P. aeruginosa* intrinsically produces chromosomal AmpC cephalosporinases, and has acquired both narrow-spectrum (such as PSE-1 and PSE-4) and broad-spectrum (PER-1, VEB-1, BEL-1, GES-1, and GES-13) β-lactamases [223]. All these enzymes do not confer resistance to carbapenems. Carbapenemases in *P. aeruginosa* belong to Ambler classes A, B, and D and have been extensively investigated. It is worth mentioning that very peculiar, naturally occurring, chromosomally encoded, class C enzymes that confer weak carbapenem-hydrolyzing activity have been identified in *P. aeruginosa*. The clinical relevance of these enzymes remains to be clarified [224].

Among class A carbapenemases, GES-2 was the first carbapenemase of the group to be identified in a strain of *P. aeruginosa* isolated from a patient in South Africa [65]. The gene encoding GES-2 was located on a self-transferrable plasmid, and differed from GES-1 by one amino acid substitution, which was thought to extend its substrate profile to hydrolyze imipenem. Recently, GES-5 and GES-24 carbapenemases were isolated from *P. aeruginosa* from long-term care facilities in South Korea [225]. GES-5 was also detected in studies from Canada [226], Indonesia [227], Dubai [67], and Japan [228]. A variant of GES-5 called GES-18 was isolated from *P. aeruginosa* recovered from the endotracheal aspirate of an elderly patient hospitalized in Belgium [229]. Both GES-5 and GES-18 genes are chromosomally encoded and were shown to be parts of class 1 integrons. Likewise, the chromosomal *bla*_GES-20_ carbapenemase gene was identified in a study of hospital isolates of *P. aeruginosa* from Mexico [68]. Apart from GES family, and, although KPC is commonly detected in *Enterobacteriaceae*, reports of KPC-2 in *P. aeruginosa* are accumulating, including data from Germany [230], Brazil [231], China [232], and Puerto Rico [233]. The *bla*_KPC_ gene is carried by plasmids, which suggests the possibility of an inter-genus spread from *Enterobacteriaceae* into non-fermenters, even though evidence of such a spread is still unclear [224].

Despite existence of the above class A carbapenemases, MBLs remain the primary carbapenemases produced by *P. aeruginosa*. Evidence exists that MBL genes were first propagated in pseudomonads, especially *P. aeruginosa*, before appearing in *Enterobacteriaceae*, including *S. marcescens*, *K. pneumoniae*, *C. freundii*, *E. coli*, and *Enterobacter* spp. [73]. As described earlier (Section 4), MBLs of types IMP, VIM, SPM, and GIM are widely distributed in *P. aeruginosa* worldwide, and it can be considered the main reservoir for these enzymes [219]. For example, out of more than 50 variants of IMP, 32 have been reported in *P. aeruginosa* including IMP-1, IMP-2, IMP-4, IMP-5, IMP-6, IMP-7, IMP-10, IMP-13, IMP-19, IMP-20, and others [224].

The first NDM-1 producing *P. aeruginosa* was reported in 2011 in Serbia, and this was simultaneously the first incidence of NDM-1 from the Balkan region [234]. Soon thereafter, a report described acute pyelonephritis due to NDM-1 producing *P. aeruginosa* in a patient in France who was previously hospitalized in Serbia [235]. Since then, additional reports of NDM-1-producing *P. aeruginosa* have appeared from Iraq [236], Poland [237], Singapore [238], and Malaysia [239]. As a reflection on diversity of MBLs encountered in *P. aeruginosa* in clinical settings, another MBL with 40% similarity to NDM-type enzymes, was isolated in Florence, Italy, from a *P. aeruginosa* isolate recovered from a patient with a vascular graft infection [240]. The enzyme was named FIM-1, (Florence IMipenemase), and the *bla*_FIM-1_ gene was apparently inserted into the chromosome and associated with the IS*CR19* element. Most recently in 2019, and concerning MBL detection in *P. aeruginosa*, Boyd and colleagues from Canada [241] used whole-genome sequencing (WGS) to identify carbapenemase production in four clinical isolates of *P. aeruginosa* that were PCR-negative for KPC, OXA-48, NDM, VIM, IMP, GES, and NMC/IMI carbapenemase genes. The WGS analysis revealed a novel MBL gene, *bla*_CAM-1_ (Central Alberta MB-1L), chromosomally located in a 73 kb integrative element, which was not transferrable by conjugation.

The last group of carbapenemases that may be harbored by *P. aeruginosa* includes the class D enzymes. Although this organism naturally encodes OXA-50, and many expanded spectrum OXA enzymes like OXA-11, OXA-13, OXA-14, OXA-16, OXA-19, OXA-31, OXA-36, OXA-128, OXA-142, OXA-145, and OXA-183 [224]. All these enzyme varieties do not compromise carbapenem activity. The known OXA enzymes from *P. aeruginosa* that act as carbapenemases include OXA-40 and OXA-198. OXA-40 carbapenemase was detected in two isolates of *P. aeruginosa* resistant to imipenem in Spain in 2006. Sequence analysis showed the plasmid-encoded gene had 100% homology with the gene previously described in *A. baumannii* [242]. On the other hand, OXA-198 carbapenemase was isolated from *P. aeruginosa* recovered from a patient with ventilator-associated pneumonia in Belgium in 2011 [243]. New investigations on *P. aeruginosa* revealed OXA-23, OXA-24/40, and OXA-58, which is commonly produced by A*. baumannii*. This provides the basis to further elucidate oxacillinases in *P. aeruginosa* [244,245].

### 7.3. Acinetobacter

The first decade of the 20th century has seen a surge in the incidence of infections due to several highly antimicrobial-resistant bacteria in hospitals worldwide. *Acinetobacter* species, especially *A. baumannii*, is one such organism that turned from an occasional respiratory pathogen into a major nosocomial one [246]. The ability of this pathogen to gain several virulence factors and to survive for prolonged periods has led to its successful emergence as an opportunistic pathogen causing bacteremia, sepsis, meningitis, and urinary tract infections. Additionally, *A. baumannii* has a remarkable propensity for rapid acquisition of resistance to an extensive range of antimicrobial agents. It exhibits a major resistance profile toward carbapenems and other *β*-lactams, which leaves clinicians with limited therapeutic options [247]. Carbapenem resistance among *A. baumannii* is conferred by several coexisting mechanisms including a decrease in outer membrane permeability, efflux pumps, hyperproduction of AmpC cephalosporinases, and modification of penicillin-binding proteins. However, the most prevalent mechanism of carbapenem resistance among *A. baumannii* is associated with carbapenemases of Ambler classes B and D. In addition, there have also been reports of resistance mediated by selected Ambler class A carbapenemases among *A. baumannii* strains [248].

Ambler class B enzymes have been rising in *A. baumannii* for the previous decade [246]. Perhaps the most concerning is NDM, which has been identified in *A. baumannii* since 2010 in India, where it co-existed with OXA-23 [249]. In 2011, 4 NDM-1-producing isolates were identified in a multicenter surveillance study in China [250]. In 2013, NDM-1-producing *A. baumannii* caused a hospital outbreak in France, which was traced to two index patients previously hospitalized in Algeria, and with no clear link to the Indian subcontinent [251]. Given the relationship between North African countries and European countries, it was then anticipated that the spread of NDM-1-producing *A. baumannii* would occur rapidly, as this organism is difficult to eradicate. Expectedly, similar isolates were detected in Greece [252], Denmark [253], Belgium [254], and Czech Republic [255]. NDM-1-producing *A. baumannii* were then considered disseminated in European countries and classified into three distinct sequence types harboring a chromosomally located *bla*_NDM-1_ gene within a Tn*125* transposon [256]. NDM-1 was also isolated from *A. baumannii* recovered from Syrian civilians injured during the civil war [99]. NDM-2-producing *A. baumannii* was detected in UAE [257] and Egypt [100], which raises the suspicion that, besides the Indian subcontinent, the Middle East area may also present a reservoir for NDM-producing *A. baumannii*. Sporadically, about nine IMP variants and five VIM variants were detected in *A. baumannii* [224]. SIM-1 has been reported from *A. baumannii* in South Korea were it appears to be widespread [120], and has disseminated to other *Acinetobacter* species like *A. pittii* and *Acinetobacter nosocomialis* [258].

*A. baumannii* possesses naturally occurring class D β-lactamases, known as OXA-51-like enzymes, that exhibit weak carbapenemase activity. Noticeably, the corresponding genes are not expressed or only weakly expressed in most isolates. However, once overexpressed, they may subsequently be involved in reduced susceptibility to carbapenems [224]. The overexpression *bla*_OXA-51-like_ genes is often driven by the upstream insertion of an IS*Aba1* element, which provides strong promoter sequences, and increases expression of OXA-51 by about eight-fold [135].

Besides the naturally occurring *bla*_OXA-51_, many acquired class D carbapenemases have been detected in *A. baumannii*. Currently, perhaps the most important OXA-type enzyme highly prevalent in this organism is OXA-23. As of 2010, worldwide dissemination of the *bla*_OXA-23_ gene was established after a study of strains from 15 countries in different regions and on five continents [28]. The contemporary worldwide dissemination of this gene was shown to be driven by >7 MLST types associated with different genetic structures and located on either chromosomes or plasmids. The complex and dynamic spreading of *bla*_OXA-23_ will be difficult to control because this spread is not associated with a single genetic entity. The transposons Tn*2006*, Tn*2007*, and Tn*2008* were identified as genetic structures harboring this gene. In Tn*2006*, the *bla*_OXA-23_ gene is flanked by two copies of the insertion sequence IS*Aba1*, which are located on opposite orientations [259]. Tn*2008* is similar to Tn*2006* but lacks the second copy of IS*Aba1* and the *bla*_OXA-23_ gene is associated with one copy of IS*Aba4* (which differs from IS*Aba1*) in Tn2007 [132]. While OXA-23-like enzymes are commonly associated with hospital outbreaks, and pandemic clones producing these enzymes are identified [260,261], other families like OXA-58, OXA-143, and OXA-235 are identified on a more limited or sporadic basis, as explained above in Section 5. *bla*_OXA-23_ has been detected in *A. baumannii* from companion animals at veterinary clinics in Germany, on plasmids with Tn*2008* [262]. Therefore, resistance determinants and clonal lineages of *A. baumannii* strains globally emerging in humans require close molecular surveillance to monitor this interspecies spread and mitigate its effect on both human and non-human hosts.

Among carbapenemases of Ambler class A, specific GES variants identified in *A. baumannii* compromise carbapenem activity. Among these are GES-5 [263] detected in Saudi Arabia, GES-11 detected in France [69] and Lebanon [83], and GES-14 detected in France [264] and Kuwait [265]. KPC enzymes, belonging to Ambler class A, have mostly been identified in *Enterobacteriaceae*, but a report from Puerto Rico in 2010 described KPC-2, KPC-3, KPC-4, and KPC-10 in *A. baumannii* [266]. Soon thereafter, molecular characterization of *bla*_KPC_ in *A. baumannii* in the same country revealed its association with the transposon Tn*4401b* and its integration in the chromosome by a transposition event mediated by the transposase IS*Ecp1* [267]. Further analysis showed that the gene is associated with a plasmid fragment derived from *Enterobacteriaceae* [59]. KPC-3 was detected in *A. baumannii* from an infected wound culture of a patient admitted to a university hospital in Portugal. The isolate accumulated carbapenem, tigecycline, and colistin resistance [60]. No other reports of KPC-producing *A. baumannii* exist in other countries.

Regarding the class C enzymes, intrinsic to *A. baumannii*, exists as a chromosomal AmpC β-lactamase, that, like OXA-51, is normally expressed only at a low level. However, it can be overexpressed as a result of upstream insertion of IS*Aba1* sequences, which exist in up to 13 copies per cell, and is thought to act as a moving switch to the nearby genes [181]. WGS experiments have shown the existence of multiple variants of AmpC, called *Acinetobacter*-derived cephalosporinases (ADCs) [268]. Although insertion of IS*Aba1* upstream of the genes encoding ADCs leads to a likely higher β-lactam hydrolysis rate, the clinical effect of such a factor on carbapenem resistance is not clear [224]. Nevertheless, in 2014, Jeon et al. [179] defined a novel ADC, named ADC-68. The gene encoding this enzyme was chromosomal, and analysis of the enzyme structure showed a particular conformation that was able to accommodate not only extended-spectrum cepahlosporins, but also carbapenems. Therefore, ADC-68 was the first reported enzyme among chromosomal class C β-lactamases to possess both extended-spectrum β-lactamase and carbapenemase activities.

### 7.4. Other Gram-Negative Organisms

Besides the above clinically significant bacteria, carbapenemases are also detected in other Gram-negative organisms both from patients and from the environment. For instance, the class A carbapenemase, PenA, was detected in *Burkholderia cepacia* complex isolates from cystic fibrosis patients [269]. The environment plays a major role in the maintenance and genetic exchange of resistance determinants between environmental and pathogenic bacteria. This explains why carbapenemase studies on aquatic sources (rivers, lakes, and wastewater treatment plants), livestock, and wildlife are accumulating. For example, hospital wastewater treatment plants in China were shown to contain KPC-2-producing *Raoultella ornithinolytica* [270], which is an aquatic Gram-negative organism with emerging importance in hospital infections associated with invasive procedures [271]. According to a Swiss study in 2019, the environmental Gram-negative organism *Zhongshania aliphaticivorans*, which lives in marine sediments, was shown to produce ZHO-1. This is an intrinsic carbapenemase with significant hydrolytic activity against most β-lactams including penicillins, cephalosporins, and carbapenems, with the exception of aztreonam and cefepime [272]. Another Swiss study, also in 2019, recognized the environmental Gram-negative species *Pseudobacteriovorax antillogorgiicola* to harbor PAN-1, which is an MBL with hydrolytic activity toward most β-lactams including carbapenems but not cefepime and aztreonam [273]. Results from the last two studies further add to the knowledge that environmental species are a reservoir of possible clinically relevant MBLs. Xin et al. reported pollution of estuarine water with bacterial genera including *Rheinheimera*, *Stenotrophomonas*, *Shewanella*, *Raoultella*, *Vibrio*, *Pseudoalteromonas*, *Algoriphagus*, *Bowmanella,* and *Thalassospira*, which all harbor *bla*_OXA-58_ [155] conferring resistance to carbapenems and penicillin and an ability to hydrolyze cefpirome and cephalothin but not ceftazidime, cefotaxime, or cefepime.

## 8. Conclusions

Almost three decades after their original discovery in Gram-negative bacteria, carbapenemases linger to create a definite growing threat to public health, and carbapenemase-encoding genes are already widespread in many parts of the world. Awareness of the prevalence and incidence of carbapenemases is crucial in preventing their spread and selection of appropriate prevention and containment options. Perhaps particularly alarming is the fact that carbapenemases are not only restricted to hospital isolates where they compromise advanced medicine. They have been continuously circulating among hospitals, long-term care facilities, community, animals, and the environment. In a period of widespread international travel, tourism, population migration, and patient transfer to receive medical care, the association between a specific resistance mechanism and a given region or country may change, which creates an urgent need for routine local, national, and global surveillance of carbapenemases. Furthermore, in light of dissemination of carbapenemases among both fermenters and non-fermenters, the complexity of their genetic transfer, and heterogeneity of their genetic backgrounds, ongoing research on their molecular epidemiology is imperative.

## Figures and Tables

**Table 1:** Examples of carbapenemases with respective families, initial species, country of description, and genetic location.

Ambler Carbapenemase Group	Enzyme Family	Representative Enzymes with Carbapenemase Activity	Country of Initial Detection	Species of Initial Detection	Location	Reference
**Group A**	NmcA	NmcA	France	*Enterobacter cloacae*	C	[11,12]
	SME	SME-1	UK	*Serratia marcescens*	C	[42]
		SME-4	Argentina	*S. marcescens*	C	[46]
	IMI	IMI-1	USA	*E. cloacae*	C	[47]
		IMI-2	USA	*Enterobacter asburiae*	P	[45]
		IMI-3	Hong Kong	*E. cloacae*	P	[45]
		IMI-5	Canada	*E. cloacae*	P	[48]
		IMI-6	Canada	*E. cloacae*	P	[48]
	KPC	KPC-1	USA	*Klebsiella pneumoniae*	P	[21]
		KPC-2	USA	*K. pneumoniae*	P	[49]
		KPC-3	USA	*K. pneumoniae*	P	[49,50]
	Specific varieties of GES	GES-2	South Africa	*Pseudomonas aeruginosa*	P	[65]
		GES-5	Spain	*P. aeruginosa*	C	[66]
		GES-11	France	*Acinetobacter baumannii*	P	[69]
		GES-20	Mexico	*P. aeruginosa*	C	[70]
**Group B**	IMP	IMP-1	Japan	*P. aeruginosa*	P	[13]
		IMP-2	Italy	*A. baumannii*	P	[81]
		IMP-4	USA	*K. pneumoniae*	P	[82]
		IMP-7	Australia	*P. aeruginosa*	P	[84]
	VIM	VIM-1-like	Italy	*P. aeruginosa*	P	[16]
		VIM-2-like	France	*P. aeruginosa*	P	[87]
		VIM-7-like	USA	*P. aeruginosa*	P	[89]
	NDM	NDM-1	India	*K. pneumoniae*	P	[31]
		NDM-2	Egypt	*A. baumannii*	C	[100]
		NDM-3	Japan	*Escherichia coli*	P	[101]
		NDM-4	India	*E. coli*	P	[102]
		NDM-5	UK	*E. coli*	P	[103]
**Emerging group C**	ACT	ACT-28	France	*E. cloacae*	C	[177]
	CMY	CMY-10	Korea	*E. cloacae*	P	[178]
	ADC	ADC-68	Korea	*A. baumannii*	C	[179]
**Group D**	OXA	OXA-23	Scotland	*A. baumannii*	P	[29]
		OXA-24/40	Spain	*A. baumannii*	C, P	[137,140,141]
		OXA-58	France	*A. baumannii*	P	[145]
		OXA-143	Brazil	*A. baumannii*	P	[156]
		OXA-235	USA and Mexico	*A. baumannii*	C, P	[161]
		OXA-48	Turkey	*K. pneumoniae*	C, P	[25,165,166,167]

NmcA = not metalloenzyme carbapenemase A. SME = *Serratia marcescens* enzyme. IMI = imipenem-hydrolyzing β-lactamase. KPC = *Klebsiella pneumoniae* carbapenemase. GES = Guiana extended spectrum. IMP = imipenemase. VIM = Verona- intergon-encoded metallo-β-lactamase. NDM = New Delhi metallo-β-lactamase. ACT = AmpC type. CMY = cephamycinase. ADC = *Acinetobacter*-derived cephalosporinase. OXA = oxacillinase. C = Chromosomal. P = Plasmid-encoded.

**Table 2:** Examples of carbapenemases detected in Gram-negative bacteria from animal and environmental samples.

Environmental or Animal Source		Bacterial Species	Detected Carbapenemase	Reference
Animals	Poultry farm	***Escherichia coli***	NDM-5	[98]
	Cats	***Salmonella enterica*** **serovar** ***Typhimurium***	IMP-4	[85]
	Wild mammals and birds	**Multiple** ***Enterobacteriaceae*** **species**	OXA-48	[172]
	Companion animals	***Acinetobacter baumannii***	OXA-48	[173]
			OXA-23	[262]
	Cattle	***A. baumannii***	OXA-24	[143]
	Domestic parrot	***A. baumannii***	OXA-72	[144]
Water	Wastewater	***E. coli***	KPC-2	[63]
		***Citrobacter freundii***	OXA-204	[171]
	Hospital wastewater	***E. coli***	KPC-2	[64]
		***Raoltella ornitholitica***	KPC-2	[270]
		***Acinetobacter towneri***	OXA-58	[154]
	Coastal water	***A. towneri***	OXA-58	[155]
	Municipal water	***Acinetobacter lwoffii***	OXA-235 and OXA-278	[163]
	River water	***Klebsiella pneumoniae***	KPC-2	[216]
	Estuarine water	**Multiple Gram-negative environmental species**	OXA-58	[155]
	Lake water	***Pseudomonas otitidis***	POM-1	[97]
Environmental samples	Marine sediments	***Zhongshania aliphaticivorans***	ZHO-1	[272]
	Soft coral	***Pseudobacteriovorax antillogorgiicola***	PAN-1	[273]

NDM = New Delhi metallo-β-lactamase. IMP = imipenemase. OXA = oxacillinase. KPC = *Klebsiella pneumoniae* carbapenemase. POM = Pseudomonas otitidis metallo-β-lactamase. ZHO = *Zhongshania aliphaticivorans* carbapenemase. PAN = *Pseudobacteriovorax antillogorgiicola* carbapenemase.

## References

[1] Jean S.-S., Lee W.-S., Lam C., Hsu C.-W., Chen R.-J., Hsueh P.-R. (2015). Carbapenemase-producing Gram-negative bacteria: Current epidemics, antimicrobial susceptibility and treatment options. Future Microbiol..

[2] Bonomo R.A., Burd E.M., Conly J., Limbago B.M., Poirel L., Segre J.A., Westblade L.F. (2018). Carbapenemase-Producing Organisms: A Global Scourge. Clin. Infect. Dis. Off. Publ. Infect. Dis. Soc. Am..

[3] Kanj S.S., Kanafani Z.A. (2011). Current concepts in antimicrobial therapy against resistant gram-negative organisms: Extended-spectrum beta-lactamase-producing Enterobacteriaceae, carbapenem-resistant Enterobacteriaceae, and multidrug-resistant *Pseudomonas aeruginosa*. Mayo Clin. Proc..

[4] Bush K. (2018). Past and Present Perspectives on β-Lactamases. Antimicrob. Agents Chemother..

[5] Touati A., Mairi A. (2019). Carbapenemase-Producing Enterobacterales in Algeria: A Systematic Review. Microb. Drug Resist. Larchmt. N.

[6] Carfi A., Pares S., Duée E., Galleni M., Duez C., Frère J.M., Dideberg O. (1995). The 3-D structure of a zinc metallo-beta-lactamase from *Bacillus cereus* reveals a new type of protein fold. EMBO J..

[7] Duval V., Swinnen M., Lepage S., Brans A., Granier B., Franssen C., Frère J.-M., Joris B. (2003). The kinetic properties of the carboxy terminal domain of the *Bacillus licheniformis* 749/I BlaR penicillin-receptor shed a new light on the derepression of beta-lactamase synthesis. Mol. Microbiol..

[8] Queenan A.M., Bush K. (2007). Carbapenemases: The versatile beta-lactamases. Clin. Microbiol. Rev..

[9] Cullmann W., Dick W. (1990). Heterogeneity of beta-lactamase production in *Pseudomonas maltophilia*, a nosocomial pathogen. Chemotherapy.

[10] Yang Y.J., Wu P.J., Livermore D.M. (1990). Biochemical characterization of a beta-lactamase that hydrolyzes penems and carbapenems from two *Serratia marcescens* isolates. Antimicrob. Agents Chemother..

[11] Nordmann P., Mariotte S., Naas T., Labia R., Nicolas M.H. (1993). Biochemical properties of a carbapenem-hydrolyzing beta-lactamase from *Enterobacter cloacae* and cloning of the gene into *Escherichia coli*. Antimicrob. Agents Chemother..

[12] Naas T., Nordmann P. (1994). Analysis of a carbapenem-hydrolyzing class A beta-lactamase from *Enterobacter cloacae* and of its LysR-type regulatory protein. Proc. Natl. Acad. Sci. USA.

[13] Watanabe M., Iyobe S., Inoue M., Mitsuhashi S. (1991). Transferable imipenem resistance in *Pseudomonas aeruginosa*. Antimicrob. Agents Chemother..

[14] Tsakris A., Pournaras S., Woodford N., Palepou M.F., Babini G.S., Douboyas J., Livermore D.M. (2000). Outbreak of infections caused by *Pseudomonas aeruginosa* producing VIM-1 carbapenemase in Greece. J. Clin. Microbiol..

[15] Cardoso O., Leitão R., Figueiredo A., Sousa J.C., Duarte A., Peixe L.V. (2002). Metallo-beta-lactamase VIM-2 in clinical isolates of *Pseudomonas aeruginosa* from Portugal. Microb. Drug Resist. Larchmt. N.

[16] Lauretti L., Riccio M.L., Mazzariol A., Cornaglia G., Amicosante G., Fontana R., Rossolini G.M. (1999). Cloning and characterization of blaVIM, a new integron-borne metallo-beta-lactamase gene from a *Pseudomonas aeruginosa* clinical isolate. Antimicrob. Agents Chemother..

[17] Crespo M.P., Woodford N., Sinclair A., Kaufmann M.E., Turton J., Glover J., Velez J.D., Castañeda C.R., Recalde M., Livermore D.M. (2004). Outbreak of carbapenem-resistant *Pseudomonas aeruginosa* producing VIM-8, a novel metallo-beta-lactamase, in a tertiary care center in Cali, Colombia. J. Clin. Microbiol..

[18] Gales A.C., Menezes L.C., Silbert S., Sader H.S. (2003). Dissemination in distinct Brazilian regions of an epidemic carbapenem-resistant *Pseudomonas aeruginosa* producing SPM metallo-beta-lactamase. J. Antimicrob. Chemother..

[19] Walsh T.R. (2005). The emergence and implications of metallo-beta-lactamases in Gram-negative bacteria. Clin. Microbiol. Infect..

[20] Pournaras S., Maniati M., Petinaki E., Tzouvelekis L.S., Tsakris A., Legakis N.J., Maniatis A.N. (2003). Hospital outbreak of multiple clones of *Pseudomonas aeruginosa* carrying the unrelated metallo-beta-lactamase gene variants blaVIM-2 and blaVIM-4. J. Antimicrob. Chemother..

[21] Yigit H., Queenan A.M., Anderson G.J., Domenech-Sanchez A., Biddle J.W., Steward C.D., Alberti S., Bush K., Tenover F.C. (2001). Novel carbapenem-hydrolyzing beta-lactamase, KPC-1, from a carbapenem-resistant strain of *Klebsiella pneumoniae*. Antimicrob. Agents Chemother..

[22] Yigit H., Queenan A.M., Rasheed J.K., Biddle J.W., Domenech-Sanchez A., Alberti S., Bush K., Tenover F.C. (2003). Carbapenem-resistant strain of *Klebsiella oxytoca* harboring carbapenem-hydrolyzing beta-lactamase KPC-2. Antimicrob. Agents Chemother..

[23] Lee C.-R., Lee J.H., Park K.S., Kim Y.B., Jeong B.C., Lee S.H. (2016). Global Dissemination of Carbapenemase-Producing *Klebsiella pneumoniae*: Epidemiology, Genetic Context, Treatment Options, and Detection Methods. Front. Microbiol..

[24] Porreca A.M., Sullivan K.V., Gallagher J.C. (2018). The Epidemiology, Evolution, and Treatment of KPC-Producing Organisms. Curr. Infect. Dis. Rep..

[25] Poirel L., Héritier C., Tolün V., Nordmann P. (2004). Emergence of oxacillinase-mediated resistance to imipenem in *Klebsiella pneumoniae*. Antimicrob. Agents Chemother..

[26] Potron A., Poirel L., Nordmann P. (2014). Derepressed transfer properties leading to the efficient spread of the plasmid encoding carbapenemase OXA-48. Antimicrob. Agents Chemother..

[27] Potron A., Poirel L., Rondinaud E., Nordmann P. (2013). Intercontinental spread of OXA-48 beta-lactamase-producing Enterobacteriaceae over a 11-year period, 2001 to 2011. Eurosurveillance.

[28] Mugnier P.D., Poirel L., Naas T., Nordmann P. (2010). Worldwide dissemination of the blaOXA-23 carbapenemase gene of *Acinetobacter baumannii*. Emerg. Infect. Dis..

[29] Scaife W., Young H.K., Paton R.H., Amyes S.G. (1995). Transferable imipenem-resistance in *Acinetobacter* species from a clinical source. J. Antimicrob. Chemother..

[30] Donald H.M., Scaife W., Amyes S.G., Young H.K. (2000). Sequence analysis of ARI-1, a novel OXA beta-lactamase, responsible for imipenem resistance in *Acinetobacter baumannii* 6B92. Antimicrob. Agents Chemother..

[31] Yong D., Toleman M.A., Giske C.G., Cho H.S., Sundman K., Lee K., Walsh T.R. (2009). Characterization of a new metallo-beta-lactamase gene, bla(NDM-1), and a novel erythromycin esterase gene carried on a unique genetic structure in *Klebsiella pneumoniae* sequence type 14 from India. Antimicrob. Agents Chemother..

[32] Pittalis S., Ferarro F., Puro V. (2011). NDM-1: The superbug?. Infez. Med..

[33] Poirel L., Potron A., Nordmann P. (2012). OXA-48-like carbapenemases: The phantom menace. J. Antimicrob. Chemother..

[34] Meletis G., Chatzidimitriou D., Malisiovas N. (2015). Double- and multi-carbapenemase-producers: The excessively armored bacilli of the current decade. Eur. J. Clin. Microbiol. Infect. Dis..

[35] Meletis G. (2016). Carbapenem resistance: Overview of the problem and future perspectives. Ther. Adv. Infect. Dis..

[36] Bush K., Jacoby G.A., Medeiros A.A. (1995). A functional classification scheme for beta-lactamases and its correlation with molecular structure. Antimicrob. Agents Chemother..

[37] Bush K. (2013). The ABCD’s of β-lactamase nomenclature. J. Infect. Chemother..

[38] Bush K., Jacoby G.A. (2010). Updated functional classification of beta-lactamases. Antimicrob. Agents Chemother..

[39] Jacoby G.A. (2009). AmpC beta-lactamases. Clin. Microbiol. Rev..

[40] Bush K. (1989). Characterization of beta-lactamases. Antimicrob. Agents Chemother..

[41] Hammoudi D., Moubareck C.A., Sarkis D.K. (2014). How to detect carbapenemase producers? A literature review of phenotypic and molecular methods. J. Microbiol. Methods.

[42] Naas T., Vandel L., Sougakoff W., Livermore D.M., Nordmann P. (1994). Cloning and sequence analysis of the gene for a carbapenem-hydrolyzing class A beta-lactamase, Sme-1, from *Serratia marcescens* S6. Antimicrob. Agents Chemother..

[43] Henriques I., Moura A., Alves A., Saavedra M.J., Correia A. (2004). Molecular characterization of a carbapenem-hydrolyzing class A beta-lactamase, SFC-1, from *Serratia fonticola* UTAD54. Antimicrob. Agents Chemother..

[44] Poirel L., Héritier C., Podglajen I., Sougakoff W., Gutmann L., Nordmann P. (2003). Emergence in *Klebsiella pneumoniae* of a chromosome-encoded SHV beta-lactamase that compromises the efficacy of imipenem. Antimicrob. Agents Chemother..

[45] Naas T., Dortet L., Iorga B.I. (2016). Structural and Functional Aspects of Class A Carbapenemases. Curr. Drug Targets.

[46] Dabos L., Patiño-Navarrete R., Nastro M., Famiglietti A., Glaser P., Rodriguez C.H., Naas T. (2019). SME-4-producing *Serratia marcescens* from Argentina belonging to clade 2 of the *S. marcescens* phylogeny. J. Antimicrob. Chemother..

[47] Rasmussen B.A., Bush K., Keeney D., Yang Y., Hare R., O’Gara C., Medeiros A.A. (1996). Characterization of IMI-1 beta-lactamase, a class A carbapenem-hydrolyzing enzyme from *Enterobacter cloacae*. Antimicrob. Agents Chemother..

[48] Boyd D.A., Mataseje L.F., Davidson R., Delport J.A., Fuller J., Hoang L., Lefebvre B., Levett P.N., Roscoe D.L., Willey B.M. (2017). Enterobacter cloacae Complex Isolates Harboring blaNMC-A or blaIMI-Type Class A Carbapenemase Genes on Novel Chromosomal Integrative Elements and Plasmids. Antimicrob. Agents Chemother..

[49] Bratu S., Mooty M., Nichani S., Landman D., Gullans C., Pettinato B., Karumudi U., Tolaney P., Quale J. (2005). Emergence of KPC-possessing *Klebsiella pneumoniae* in Brooklyn, New York: Epidemiology and recommendations for detection. Antimicrob. Agents Chemother..

[50] Woodford N., Tierno P.M., Young K., Tysall L., Palepou M.-F.I., Ward E., Painter R.E., Suber D.F., Shungu D., Silver L.L. (2004). Outbreak of *Klebsiella pneumoniae* producing a new carbapenem-hydrolyzing class A beta-lactamase, KPC-3, in a New York Medical Center. Antimicrob. Agents Chemother..

[51] Deshpande L.M., Rhomberg P.R., Sader H.S., Jones R.N. (2006). Emergence of serine carbapenemases (KPC and SME) among clinical strains of Enterobacteriaceae isolated in the United States Medical Centers: Report from the MYSTIC Program (1999–2005). Diagn. Microbiol. Infect. Dis..

[52] Nordmann P., Naas T., Poirel L. (2011). Global spread of Carbapenemase-producing Enterobacteriaceae. Emerg. Infect. Dis..

[53] Van Beek J., Räisänen K., Broas M., Kauranen J., Kähkölä A., Laine J., Mustonen E., Nurkkala T., Puhto T., Sinkkonen J. (2019). Tracing local and regional clusters of carbapenemase-producing *Klebsiella pneumoniae* ST512 with whole genome sequencing, Finland, 2013 to 2018. Eurosurveillance.

[54] Giddins M.J., Macesic N., Annavajhala M.K., Stump S., Khan S., McConville T.H., Mehta M., Gomez-Simmonds A., Uhlemann A.-C. (2018). Successive Emergence of Ceftazidime-Avibactam Resistance through Distinct Genomic Adaptations in blaKPC-2-Harboring *Klebsiella pneumoniae* Sequence Type 307 Isolates. Antimicrob. Agents Chemother..

[55] Balm M.N.D., Ngan G., Jureen R., Lin R.T.P., Teo J. (2012). Molecular characterization of newly emerged blaKPC-2-producing *Klebsiella pneumoniae* in Singapore. J. Clin. Microbiol..

[56] Naas T., Cuzon G., Villegas M.-V., Lartigue M.-F., Quinn J.P., Nordmann P. (2008). Genetic structures at the origin of acquisition of the beta-lactamase bla KPC gene. Antimicrob. Agents Chemother..

[57] Galetti R., Andrade L.N., Varani A.M., Darini A.L.C. (2019). A Phage-Like Plasmid Carrying blaKPC-2 Gene in Carbapenem-Resistant *Pseudomonas aeruginosa*. Front. Microbiol..

[58] Hu Y.-Y., Wang Q., Sun Q.-L., Chen G.-X., Zhang R. (2019). A novel plasmid carrying carbapenem-resistant gene blaKPC-2 in *Pseudomonas aeruginosa*. Infect. Drug Resist..

[59] Martinez T., Martinez I., Vazquez G.J., Aquino E.E., Robledo I.E. (2016). Genetic environment of the KPC gene in *Acinetobacter baumannii* ST2 clone from Puerto Rico and genomic insights into its drug resistance. J. Med. Microbiol..

[60] Caneiras C., Calisto F., Jorge da Silva G., Lito L., Melo-Cristino J., Duarte A. (2018). First Description of Colistin and Tigecycline-Resistant *Acinetobacter baumannii* Producing KPC-3 Carbapenemase in Portugal. Antibiot. Basel Switz..

[61] Landman D., Bratu S., Quale J. (2009). Contribution of OmpK36 to carbapenem susceptibility in KPC-producing *Klebsiella pneumoniae*. J. Med. Microbiol..

[62] Zhang Y., Jiang X., Wang Y., Li G., Tian Y., Liu H., Ai F., Ma Y., Wang B., Ruan F. (2014). Contribution of β-lactamases and porin proteins OmpK35 and OmpK36 to carbapenem resistance in clinical isolates of KPC-2-producing *Klebsiella pneumoniae*. Antimicrob. Agents Chemother..

[63] Yang F., Huang L., Li L., Yang Y., Mao D., Luo Y. (2017). Discharge of KPC-2 genes from the WWTPs contributed to their enriched abundance in the receiving river. Sci. Total Environ..

[64] Ekwanzala M.D., Budeli P., Dewar J.B., Kamika I., Momba M.N.B. (2019). Draft Genome Sequences of Novel Sequence Type 3559 Carbapenem-Resistant *Klebsiella pneumoniae* Isolates Recovered from the Environment. Microbiol. Resour. Announc..

[65] Poirel L., Weldhagen G.F., Naas T., De Champs C., Dove M.G., Nordmann P. (2001). GES-2, a class A beta-lactamase from *Pseudomonas aeruginosa* with increased hydrolysis of imipenem. Antimicrob. Agents Chemother..

[66] Viedma E., Juan C., Acosta J., Zamorano L., Otero J.R., Sanz F., Chaves F., Oliver A. (2009). Nosocomial spread of colistin-only-sensitive sequence type 235 *Pseudomonas aeruginosa* isolates producing the extended-spectrum beta-lactamases GES-1 and GES-5 in Spain. Antimicrob. Agents Chemother..

[67] Ayoub Moubareck C., Hammoudi Halat D., Akkawi C., Nabi A., AlSharhan M.A., AlDeesi Z.O., Peters C.C., Celiloglu H., Karam Sarkis D. (2019). Role of outer membrane permeability, efflux mechanism, and carbapenemases in carbapenem-nonsusceptible *Pseudomonas aeruginosa* from Dubai hospitals: Results of the first cross-sectional survey. Int. J. Infect. Dis..

[68] Garza-Ramos U., Barrios H., Reyna-Flores F., Tamayo-Legorreta E., Catalan-Najera J.C., Morfin-Otero R., Rodríguez-Noriega E., Volkow P., Cornejo-Juarez P., González A. (2015). Widespread of ESBL- and carbapenemase GES-type genes on carbapenem-resistant *Pseudomonas aeruginosa* clinical isolates: A multicenter study in Mexican hospitals. Diagn. Microbiol. Infect. Dis..

[69] Moubareck C., Brémont S., Conroy M.-C., Courvalin P., Lambert T. (2009). GES-11, a novel integron-associated GES variant in *Acinetobacter baumannii*. Antimicrob. Agents Chemother..

[70] Hammoudi D., Ayoub Moubareck C., Aires J., Adaime A., Barakat A., Fayad N., Hakime N., Houmani M., Itani T., Najjar Z. (2014). Countrywide spread of OXA-48 carbapenemase in Lebanon: Surveillance and genetic characterization of carbapenem-non-susceptible Enterobacteriaceae in 10 hospitals over a one-year period. Int. J. Infect. Dis..

[71] Van Duin D., Doi Y. (2017). The global epidemiology of carbapenemase-producing Enterobacteriaceae. Virulence.

[72] Jeon J.H., Lee J.H., Lee J.J., Park K.S., Karim A.M., Lee C.-R., Jeong B.C., Lee S.H. (2015). Structural basis for carbapenem-hydrolyzing mechanisms of carbapenemases conferring antibiotic resistance. Int. J. Mol. Sci..

[73] Walsh T.R., Toleman M.A., Poirel L., Nordmann P. (2005). Metallo-beta-lactamases: The quiet before the storm?. Clin. Microbiol. Rev..

[74] Kuwabara S., Abraham E.P. (1967). Some properties of two extracellular beta-lactamases from *Bacillus cereus* 569/H. Biochem. J..

[75] Rasmussen B.A., Kovacs E. (1991). Identification and DNA sequence of a new *Bacteroides fragilis* insertion sequence-like element. Plasmid.

[76] Podglajen I., Breuil J., Casin I., Collatz E. (1995). Genotypic identification of two groups within the species *Bacteroides fragilis* by ribotyping and by analysis of PCR-generated fragment patterns and insertion sequence content. J. Bacteriol..

[77] Podglajen I., Breuil J., Collatz E. (1994). Insertion of a novel DNA sequence, 1S1186, upstream of the silent carbapenemase gene cfiA, promotes expression of carbapenem resistance in clinical isolates of *Bacteroides fragilis*. Mol. Microbiol..

[78] Diene S.M., Rolain J.-M. (2014). Carbapenemase genes and genetic platforms in Gram-negative bacilli: *Enterobacteriaceae*, *Pseudomonas* and *Acinetobacter* species. Clin. Microbiol. Infect..

[79] Leiros H.-K.S., Skagseth S., Edvardsen K.S.W., Lorentzen M.S., Bjerga G.E.K., Leiros I., Samuelsen Ø. (2014). His224 alters the R2 drug binding site and Phe218 influences the catalytic efficiency of the metallo-β-lactamase VIM-7. Antimicrob. Agents Chemother..

[80] Cornaglia G., Riccio M.L., Mazzariol A., Lauretti L., Fontana R., Rossolini G.M. (1999). Appearance of IMP-1 metallo-beta-lactamase in Europe. Lancet Lond. Engl..

[81] Riccio M.L., Franceschini N., Boschi L., Caravelli B., Cornaglia G., Fontana R., Amicosante G., Rossolini G.M. (2000). Characterization of the metallo-beta-lactamase determinant of *Acinetobacter baumannii* AC-54/97 reveals the existence of bla(IMP) allelic variants carried by gene cassettes of different phylogeny. Antimicrob. Agents Chemother..

[82] Limbago B.M., Rasheed J.K., Anderson K.F., Zhu W., Kitchel B., Watz N., Munro S., Gans H., Banaei N., Kallen A.J. (2011). IMP-producing carbapenem-resistant *Klebsiella pneumoniae* in the United States. J. Clin. Microbiol..

[83] Hammoudi Halat D., Moubareck C.A., Sarkis D.K. (2017). Heterogeneity of Carbapenem Resistance Mechanisms among Gram-Negative Pathogens in Lebanon: Results of the First Cross-Sectional Countrywide Study. Microb. Drug Resist. Larchmt. N.

[84] McCarthy K.L., Jennison A., Wailan A.M., Paterson D.L. (2017). Draft Genome Sequence of an IMP-7-Producing *Pseudomonas aeruginosa* Bloodstream Infection Isolate from Australia. Genome Announc..

[85] Abraham S., O’Dea M., Trott D.J., Abraham R.J., Hughes D., Pang S., McKew G., Cheong E.Y.L., Merlino J., Saputra S. (2016). Isolation and plasmid characterization of carbapenemase (IMP-4) producing *Salmonella enterica* Typhimurium from cats. Sci. Rep..

[86] Mollenkopf D.F., Stull J.W., Mathys D.A., Bowman A.S., Feicht S.M., Grooters S.V., Daniels J.B., Wittum T.E. (2017). Carbapenemase-Producing Enterobacteriaceae Recovered from the Environment of a Swine Farrow-to-Finish Operation in the United States. Antimicrob. Agents Chemother..

[87] Poirel L., Naas T., Nicolas D., Collet L., Bellais S., Cavallo J.D., Nordmann P. (2000). Characterization of VIM-2, a carbapenem-hydrolyzing metallo-beta-lactamase and its plasmid- and integron-borne gene from a *Pseudomonas aeruginosa* clinical isolate in France. Antimicrob. Agents Chemother..

[88] Mojica M.F., Bonomo R.A., Fast W. (2016). B1-Metallo-β-Lactamases: Where Do We Stand?. Curr. Drug Targets.

[89] Toleman M.A., Rolston K., Jones R.N., Walsh T.R. (2004). blaVIM-7, an evolutionarily distinct metallo-beta-lactamase gene in a *Pseudomonas aeruginosa* isolate from the United States. Antimicrob. Agents Chemother..

[90] Balero de Paula S., Cayô R., Streling A.P., Silva Nodari C., Pereira Matos A., Eches Perugini M.R., Gales A.C., Carrara-Marroni F.E., Yamada-Ogatta S.F. (2017). Detection of blaVIM-7 in an extensively drug-resistant *Pseudomonas aeruginosa* isolate belonging to ST1284 in Brazil. Diagn. Microbiol. Infect. Dis..

[91] Carattoli A. (2009). Resistance plasmid families in Enterobacteriaceae. Antimicrob. Agents Chemother..

[92] Zhang H., Hao Q. (2011). Crystal structure of NDM-1 reveals a common β-lactam hydrolysis mechanism. J. Off. Publ. Fed. Am. Soc. Exp. Biol..

[93] Khan A.U., Nordmann P. (2012). Spread of carbapenemase NDM-1 producers: The situation in India and what may be proposed. Scand. J. Infect. Dis..

[94] Wu W., Feng Y., Tang G., Qiao F., McNally A., Zong Z. (2019). NDM Metallo-β-Lactamases and Their Bacterial Producers in Health Care Settings. Clin. Microbiol. Rev..

[95] Poirel L., Hombrouck-Alet C., Freneaux C., Bernabeu S., Nordmann P. (2010). Global spread of New Delhi metallo-β-lactamase 1. Lancet Infect. Dis..

[96] Moghadampour M., Salari-Jazi A., Faghri J. (2018). High rate of carbapenem-resistant *Klebsiella pneumoniae* detected from hospital equipments in Iran. Acta Microbiol. Immunol. Hung..

[97] Le Terrier C., Masseron A., Uwaezuoke N.S., Edwin C.P., Ekuma A.E., Olugbeminiyi F., Shettima S., Ushi S., Poirel L., Nordmann P. (2019). Wide spread of carbapenemase producers in a Nigerian environment. J. Glob. Antimicrob. Resist..

[98] Tang B., Chang J., Cao L., Luo Q., Xu H., Lyu W., Qian M., Ji X., Zhang Q., Xia X. (2019). Characterization of an NDM-5 carbapenemase-producing *Escherichia coli* ST156 isolate from a poultry farm in Zhejiang, China. BMC Microbiol..

[99] Rafei R., Dabboussi F., Hamze M., Eveillard M., Lemarié C., Mallat H., Rolain J.-M., Joly-Guillou M.-L., Kempf M. (2014). First report of blaNDM-1-producing *Acinetobacter baumannii* isolated in Lebanon from civilians wounded during the Syrian war. Int. J. Infect. Dis..

[100] Kaase M., Nordmann P., Wichelhaus T.A., Gatermann S.G., Bonnin R.A., Poirel L. (2011). NDM-2 carbapenemase in *Acinetobacter baumannii* from Egypt. J. Antimicrob. Chemother..

[101] Tada T., Miyoshi-Akiyama T., Shimada K., Kirikae T. (2014). Biochemical analysis of metallo-β-lactamase NDM-3 from a multidrug-resistant Escherichia coli strain isolated in Japan. Antimicrob. Agents Chemother..

[102] Nordmann P., Boulanger A.E., Poirel L. (2012). NDM-4 metallo-β-lactamase with increased carbapenemase activity from *Escherichia coli*. Antimicrob. Agents Chemother..

[103] Hornsey M., Phee L., Wareham D.W. (2011). A novel variant, NDM-5, of the New Delhi metallo-β-lactamase in a multidrug-resistant *Escherichia coli* ST648 isolate recovered from a patient in the United Kingdom. Antimicrob. Agents Chemother..

[104] Dortet L., Jousset A., Sainte-Rose V., Cuzon G., Naas T. (2016). Prospective evaluation of the OXA-48 K-SeT assay, an immunochromatographic test for the rapid detection of OXA-48-type carbapenemases. J. Antimicrob. Chemother..

[105] Greissl C., Saleh A., Hamprecht A. (2019). Rapid detection of OXA-48-like, KPC, NDM, and VIM carbapenemases in Enterobacterales by a new multiplex immunochromatographic test. Eur. J. Clin. Microbiol. Infect. Dis..

[106] Tamma P.D., Simner P.J. (2018). Phenotypic Detection of Carbapenemase-Producing Organisms from Clinical Isolates. J. Clin. Microbiol..

[107] Toleman M.A., Simm A.M., Murphy T.A., Gales A.C., Biedenbach D.J., Jones R.N., Walsh T.R. (2002). Molecular characterization of SPM-1, a novel metallo-beta-lactamase isolated in Latin America: Report from the SENTRY antimicrobial surveillance programme. J. Antimicrob. Chemother..

[108] Cacci L.C., Chuster S.G., Martins N., Do Carmo P.R., De Carvalho Girão V.B., Nouér S.A., De Freitas W.V., De Matos J.A., De Gouveia Magalhães A.C., Pires Ferreira A.L. (2016). Mechanisms of carbapenem resistance in endemic *Pseudomonas aeruginosa* isolates after an SPM-1 metallo-β-lactamase producing strain subsided in an intensive care unit of a teaching hospital in Brazil. Mem. Inst. Oswaldo Cruz.

[109] Nascimento A.P.B., Ortiz M.F., Martins W.M.B.S., Morais G.L., Fehlberg L.C.C., Almeida L.G.P., Ciapina L.P., Gales A.C., Vasconcelos A.T.R. (2016). Intraclonal Genome Stability of the Metallo-β-lactamase SPM-1-producing *Pseudomonas aeruginosa* ST277, an Endemic Clone Disseminated in Brazilian Hospitals. Front. Microbiol..

[110] Chaves L., Tomich L.M., Salomão M., Leite G.C., Ramos J., Martins R.R., Rizek C., Neves P., Batista M.V., Amigo U. (2017). High mortality of bloodstream infection outbreak caused by carbapenem-resistant P. aeruginosa producing SPM-1 in a bone marrow transplant unit. J. Med. Microbiol..

[111] Martins W.M.B.S., Narciso A.C., Cayô R., Santos S.V., Fehlberg L.C.C., Ramos P.L., Da Cruz J.B., Gales A.C. (2018). SPM-1-producing *Pseudomonas aeruginosa* ST277 clone recovered from microbiota of migratory birds. Diagn. Microbiol. Infect. Dis..

[112] Hopkins K.L., Meunier D., Findlay J., Mustafa N., Parsons H., Pike R., Wright L., Woodford N. (2016). SPM-1 metallo-β-lactamase-producing *Pseudomonas aeruginosa* ST277 in the UK. J. Med. Microbiol..

[113] Salabi A.E., Toleman M.A., Weeks J., Bruderer T., Frei R., Walsh T.R. (2010). First report of the metallo-beta-lactamase SPM-1 in Europe. Antimicrob. Agents Chemother..

[114] Shahcheraghi F., Abbasalipour M., Feizabadi M., Ebrahimipour G., Akbari N. (2011). Isolation and genetic characterization of metallo-β-lactamase and carbapenamase producing strains of *Acinetobacter baumannii* from patients at Tehran hospitals. Iran. J. Microbiol..

[115] Andrade L.N., Woodford N., Darini A.L.C. (2014). International gatherings and potential for global dissemination of São Paulo metallo-β-lactamase (SPM) from Brazil. Int. J. Antimicrob. Agents.

[116] Toleman M.A., Bennett P.M., Walsh T.R. (2006). ISCR elements: Novel gene-capturing systems of the 21st century?. Microbiol. Mol. Biol. Rev..

[117] Zhao W.-H., Hu Z.-Q. (2015). Acquired metallo-β-lactamases and their genetic association with class 1 integrons and ISCR elements in Gram-negative bacteria. Future Microbiol..

[118] Castanheira M., Toleman M.A., Jones R.N., Schmidt F.J., Walsh T.R. (2004). Molecular characterization of a beta-lactamase gene, blaGIM-1, encoding a new subclass of metallo-beta-lactamase. Antimicrob. Agents Chemother..

[119] Girija S.A., Jayaseelan V.P., Arumugam P. (2018). Prevalence of VIM- and GIM-producing *Acinetobacter baumannii* from patients with severe urinary tract infection. Acta Microbiol. Immunol. Hung..

[120] Lee K., Yum J.H., Yong D., Lee H.M., Kim H.D., Docquier J.-D., Rossolini G.M., Chong Y. (2005). Novel acquired metallo-beta-lactamase gene, bla(SIM-1), in a class 1 integron from *Acinetobacter baumannii* clinical isolates from Korea. Antimicrob. Agents Chemother..

[121] Lü Y., Zhao S., Liang H., Zhang W., Liu J., Hu H. (2019). The first report of a novel IncHI1B blaSIM-1-carrying megaplasmid pSIM-1-BJ01 from a clinical *Klebsiella pneumoniae* isolate. Infect. Drug Resist..

[122] Poirel L., Rodríguez-Martínez J.-M., Al Naiemi N., Debets-Ossenkopp Y.J., Nordmann P. (2010). Characterization of DIM-1, an integron-encoded metallo-beta-lactamase from a *Pseudomonas stutzeri* clinical isolate in The Netherlands. Antimicrob. Agents Chemother..

[123] Sekiguchi J., Morita K., Kitao T., Watanabe N., Okazaki M., Miyoshi-Akiyama T., Kanamori M., Kirikae T. (2008). KHM-1, a novel plasmid-mediated metallo-beta-lactamase from a *Citrobacter freundii* clinical isolate. Antimicrob. Agents Chemother..

[124] Skagseth S., Christopeit T., Akhter S., Bayer A., Samuelsen Ø., Leiros H.-K.S. (2017). Structural Insights into TMB-1 and the Role of Residues 119 and 228 in Substrate and Inhibitor Binding. Antimicrob. Agents Chemother..

[125] Evans B.A., Amyes S.G.B. (2014). OXA β-lactamases. Clin. Microbiol. Rev..

[126] Walther-Rasmussen J., Høiby N. (2007). Class A carbapenemases. J. Antimicrob. Chemother..

[127] Sykes R.B., Matthew M. (1976). The beta-lactamases of gram-negative bacteria and their role in resistance to beta-lactam antibiotics. J. Antimicrob. Chemother..

[128] Hall L.M., Livermore D.M., Gur D., Akova M., Akalin H.E. (1993). OXA-11, an extended-spectrum variant of OXA-10 (PSE-2) beta-lactamase from *Pseudomonas aeruginosa*. Antimicrob. Agents Chemother..

[129] Poirel L., Figueiredo S., Cattoir V., Carattoli A., Nordmann P. (2008). Acinetobacter radioresistens as a silent source of carbapenem resistance for *Acinetobacter* spp.. Antimicrob. Agents Chemother..

[130] Kaitany K.-C.J., Klinger N.V., June C.M., Ramey M.E., Bonomo R.A., Powers R.A., Leonard D.A. (2013). Structures of the class D Carbapenemases OXA-23 and OXA-146: Mechanistic basis of activity against carbapenems, extended-spectrum cephalosporins, and aztreonam. Antimicrob. Agents Chemother..

[131] Smith C.A., Antunes N.T., Stewart N.K., Toth M., Kumarasiri M., Chang M., Mobashery S., Vakulenko S.B. (2013). Structural basis for carbapenemase activity of the OXA-23 β-lactamase from *Acinetobacter baumannii*. Chem. Biol..

[132] Corvec S., Poirel L., Naas T., Drugeon H., Nordmann P. (2007). Genetics and expression of the carbapenem-hydrolyzing oxacillinase gene blaOXA-23 in *Acinetobacter baumannii*. Antimicrob. Agents Chemother..

[133] Jiang L., Liang Y., Yao W., Ai J., Wang X., Zhao Z. (2019). Molecular epidemiology and genetic characterisation of carbapenem-resistant *Acinetobacter baumannii* isolates from Guangdong Province, South China. J. Glob. Antimicrob. Resist..

[134] Liu L.-L., Ji S.-J., Ruan Z., Fu Y., Fu Y.-Q., Wang Y.-F., Yu Y.-S. (2015). Dissemination of blaOXA-23 in Acinetobacter spp. in China: Main roles of conjugative plasmid pAZJ221 and transposon Tn2009. Antimicrob. Agents Chemother..

[135] Turton J.F., Ward M.E., Woodford N., Kaufmann M.E., Pike R., Livermore D.M., Pitt T.L. (2006). The role of ISAba1 in expression of OXA carbapenemase genes in *Acinetobacter baumannii*. FEMS Microbiol. Lett..

[136] Schultz M.B., Pham Thanh D., Tran Do Hoan N., Wick R.R., Ingle D.J., Hawkey J., Edwards D.J., Kenyon J.J., Phu Huong Lan N., Campbell J.I. (2016). Repeated local emergence of carbapenem-resistant *Acinetobacter baumannii* in a single hospital ward. Microb. Genom..

[137] Bou G., Oliver A., Martínez-Beltrán J. (2000). OXA-24, a novel class D beta-lactamase with carbapenemase activity in an *Acinetobacter baumannii* clinical strain. Antimicrob. Agents Chemother..

[138] Kuo S.-C., Huang W.-C., Huang T.-W., Wang H.-Y., Lai J.-F., Chen T.-L., Lauderdale T.-L. (2018). Molecular Epidemiology of Emerging blaOXA-23-Like- and blaOXA-24-Like-Carrying *Acinetobacter baumannii* in Taiwan. Antimicrob. Agents Chemother..

[139] Leungtongkam U., Thummeepak R., Wongprachan S., Thongsuk P., Kitti T., Ketwong K., Runcharoen C., Chantratita N., Sitthisak S. (2018). Dissemination of blaOXA-23, blaOXA-24, blaOXA-58, and blaNDM-1 Genes of *Acinetobacter baumannii* Isolates from Four Tertiary Hospitals in Thailand. Microb. Drug Resist. Larchmt. N.

[140] Todorova B., Velinov T., Ivanov I., Dobreva E., Kantardjiev T. (2014). First detection of OXA-24 carbapenemase-producing *Acinetobacter baumannii* isolates in Bulgaria. World J. Microbiol. Biotechnol..

[141] Hammoudi D., Moubareck C.A., Hakime N., Houmani M., Barakat A., Najjar Z., Suleiman M., Fayad N., Sarraf R., Sarkis D.K. (2015). Spread of imipenem-resistant *Acinetobacter baumannii* co-expressing OXA-23 and GES-11 carbapenemases in Lebanon. Int. J. Infect. Dis..

[142] Sevillano E., Valderrey C., Canduela M.J., Umaran A., Calvo F., Gallego L. (2006). Resistance to antibiotics in clinical isolates of *Pseudomonas aeruginosa*. Pathol. Biol..

[143] Pailhoriès H., Kempf M., Belmonte O., Joly-Guillou M.-L., Eveillard M. (2016). First case of OXA-24-producing *Acinetobacter baumannii* in cattle from Reunion Island, France. Int. J. Antimicrob. Agents.

[144] Klotz P., Jacobmeyer L., Stamm I., Leidner U., Pfeifer Y., Semmler T., Prenger-Berninghoff E., Ewers C. (2018). Carbapenem-resistant *Acinetobacter baumannii* ST294 harbouring the OXA-72 carbapenemase from a captive grey parrot. J. Antimicrob. Chemother..

[145] Poirel L., Marqué S., Héritier C., Segonds C., Chabanon G., Nordmann P. (2005). OXA-58, a novel class D {beta}-lactamase involved in resistance to carbapenems in *Acinetobacter baumannii*. Antimicrob. Agents Chemother..

[146] Salehi B., Ghalavand Z., Mohammadzadeh M., Maleki D.T., Kodori M., Kadkhoda H. (2019). Clonal relatedness and resistance characteristics of OXA-24 and -58 producing carbapenem-resistant *Acinetobacter baumannii* isolates in Tehran, Iran. J. Appl. Microbiol..

[147] Karampatakis T., Tsergouli K., Politi L., Diamantopoulou G., Iosifidis E., Antachopoulos C., Karyoti A., Sdougka M., Tsakris A., Roilides E. (2019). Polyclonal predominance of concurrently producing OXA-23 and OXA-58 carbapenem-resistant *Acinetobacter baumannii* strains in a pediatric intensive care unit. Mol. Biol. Rep..

[148] Cameranesi M.M., Morán-Barrio J., Limansky A.S., Repizo G.D., Viale A.M. (2018). Site-Specific Recombination at XerC/D Sites Mediates the Formation and Resolution of Plasmid Co-integrates Carrying a blaOXA-58- and TnaphA6-Resistance Module in *Acinetobacter baumannii*. Front. Microbiol..

[149] Zarrilli R., Vitale D., Di Popolo A., Bagattini M., Daoud Z., Khan A.U., Afif C., Triassi M. (2008). A plasmid-borne blaOXA-58 gene confers imipenem resistance to *Acinetobacter baumannii* isolates from a Lebanese hospital. Antimicrob. Agents Chemother..

[150] Girlich D., Bonnin R.A., Bogaerts P., De Laveleye M., Huang D.T., Dortet L., Glaser P., Glupczynski Y., Naas T. (2017). Chromosomal Amplification of the blaOXA-58 Carbapenemase Gene in a Proteus mirabilis Clinical Isolate. Antimicrob. Agents Chemother..

[151] Lange F., Pfennigwerth N., Gerigk S., Gohlke F., Oberdorfer K., Purr I., Wohanka N., Roggenkamp A., Gatermann S.G., Kaase M. (2017). Dissemination of blaOXA-58 in Proteus mirabilis isolates from Germany. J. Antimicrob. Chemother..

[152] Literacka E., Izdebski R., Baraniak A., Żabicka D., Schneider A., Urbanowicz P., Herda M., Hryniewicz W., Gniadkowski M. (2019). Proteus mirabilis Producing the OXA-58 Carbapenemase in Poland. Antimicrob. Agents Chemother..

[153] Chen Y., Guo P., Huang H., Huang Y., Wu Z., Liao K. (2019). Detection of co-harboring OXA-58 and NDM-1 carbapenemase producing genes resided on a same plasmid from an *Acinetobacter pittii* clinical isolate in China. Iran. J. Basic Med. Sci..

[154] Jiang N., Zhang X., Zhou Y., Zhang Z., Zheng X. (2019). Whole-genome sequencing of an NDM-1- and OXA-58-producing *Acinetobacter towneri* isolate from hospital sewage in Sichuan Province, China. J. Glob. Antimicrob. Resist..

[155] Xin R., Zhang K., Wu N., Zhang Y., Niu Z. (2019). The pollution level of the blaOXA-58 carbapenemase gene in coastal water and its host bacteria characteristics. Environ. Pollut..

[156] Higgins P.G., Poirel L., Lehmann M., Nordmann P., Seifert H. (2009). OXA-143, a novel carbapenem-hydrolyzing class D beta-lactamase in *Acinetobacter baumannii*. Antimicrob. Agents Chemother..

[157] Neves F.C., Clemente W.T., Lincopan N., Paião I.D., Neves P.R., Romanelli R.M., Lima S.S.S., Paiva L.F., Mourão P.H.O., Nobre-Junior V.A. (2016). Clinical and microbiological characteristics of OXA-23- and OXA-143-producing *Acinetobacter baumannii* in ICU patients at a teaching hospital, Brazil. Braz. J. Infect. Dis..

[158] Dias V.C., Resende J.A., Bastos A.N., De Andrade Bastos L.Q., De Andrade Bastos V.Q., Bastos R.V., Diniz C.G., Da Silva V.L. (2017). Epidemiological, Physiological, and Molecular Characteristics of a Brazilian Collection of Carbapenem-Resistant *Acinetobacter baumannii* and *Pseudomonas aeruginosa*. Microb. Drug Resist. Larchmt. N.

[159] Sarikhani Z., Nazari R., Nateghi Rostami M. (2017). First report of OXA-143-lactamase producing *Acinetobacter baumannii* in Qom, Iran. Iran. J. Basic Med. Sci..

[160] Gionco B., Pelayo J.S., Venancio E.J., Cayô R., Gales A.C., Carrara-Marroni F.E. (2012). Detection of OXA-231, a new variant of blaOXA-143, in *Acinetobacter baumannii* from Brazil: A case report. J. Antimicrob. Chemother..

[161] Higgins P.G., Pérez-Llarena F.J., Zander E., Fernández A., Bou G., Seifert H. (2013). OXA-235, a novel class D β-lactamase involved in resistance to carbapenems in *Acinetobacter baumannii*. Antimicrob. Agents Chemother..

[162] Boyd D.A., Mataseje L.F., Pelude L., Mitchell R., Bryce E., Roscoe D., Embree J., Katz K., Kibsey P., Lavallee C. (2019). Results from the Canadian Nosocomial Infection Surveillance Program for detection of carbapenemase-producing *Acinetobacter* spp. in Canadian hospitals, 2010–2016. J. Antimicrob. Chemother..

[163] Ng C., Tan B., Jiang X.-T., Gu X., Chen H., Schmitz B.W., Haller L., Charles F.R., Zhang T., Gin K. (2019). Metagenomic and Resistome Analysis of a Full-Scale Municipal Wastewater Treatment Plant in Singapore Containing Membrane Bioreactors. Front. Microbiol..

[164] Carrër A., Poirel L., Eraksoy H., Cagatay A.A., Badur S., Nordmann P. (2008). Spread of OXA-48-positive carbapenem-resistant *Klebsiella pneumoniae* isolates in Istanbul, Turkey. Antimicrob. Agents Chemother..

[165] Poirel L., Bonnin R.A., Nordmann P. (2012). Genetic features of the widespread plasmid coding for the carbapenemase OXA-48. Antimicrob. Agents Chemother..

[166] Turton J.F., Doumith M., Hopkins K.L., Perry C., Meunier D., Woodford N. (2016). Clonal expansion of *Escherichia coli* ST38 carrying a chromosomally integrated OXA-48 carbapenemase gene. J. Med. Microbiol..

[167] Poirel L., Abdelaziz M.O., Bernabeu S., Nordmann P. (2013). Occurrence of OXA-48 and VIM-1 carbapenemase-producing Enterobacteriaceae in Egypt. Int. J. Antimicrob. Agents.

[168] Pitout J.D.D., Peirano G., Kock M.M., Strydom K.-A., Matsumura Y. (2019). The Global Ascendency of OXA-48-Type Carbapenemases. Clin. Microbiol. Rev..

[169] Potron A., Nordmann P., Lafeuille E., Al Maskari Z., Al Rashdi F., Poirel L. (2011). Characterization of OXA-181, a carbapenem-hydrolyzing class D beta-lactamase from *Klebsiella pneumoniae*. Antimicrob. Agents Chemother..

[170] Messaoudi A., Saras E., Grami R., Bouallègue O., Boujâafar N., Madec J.-Y., Mansour W., Haenni M. (2019). Emergence of OXA-204 carbapenemase in *Enterobacter cloacae*. Int. J. Antimicrob. Agents.

[171] Sghaier S., Abbassi M.S., Pascual A., Serrano L., Díaz-De-Alba P., Said M.B., Hassen B., Ibrahim C., Hassen A., López-Cerero L. (2019). Extended-spectrum β-lactamase-producing Enterobacteriaceae from animal origin and wastewater in Tunisia: First detection of O25b-B23-CTX-M-27-ST131 *Escherichia coli* and CTX-M-15/OXA-204-producing *Citrobacter freundii* from wastewater. J. Glob. Antimicrob. Resist..

[172] Darwich L., Vidal A., Seminati C., Albamonte A., Casado A., López F., Molina-López R.A., Migura-Garcia L. (2019). High prevalence and diversity of extended-spectrum β-lactamase and emergence of OXA-48 producing Enterobacterales in wildlife in Catalonia. PLoS ONE.

[173] Pulss S., Stolle I., Stamm I., Leidner U., Heydel C., Semmler T., Prenger-Berninghoff E., Ewers C. (2018). Multispecies and Clonal Dissemination of OXA-48 Carbapenemase in Enterobacteriaceae From Companion Animals in Germany, 2009–2016. Front. Microbiol..

[174] Bae D.-W., Jung Y.-E., An Y.J., Na J.-H., Cha S.-S. (2019). Structural Insights into Catalytic Relevances of Substrate Poses in ACC-1. Antimicrob. Agents Chemother..

[175] Mammeri H., Guillon H., Eb F., Nordmann P. (2010). Phenotypic and biochemical comparison of the carbapenem-hydrolyzing activities of five plasmid-borne AmpC β-lactamases. Antimicrob. Agents Chemother..

[176] Koyano S., Saito R., Nagai R., Tatsuno K., Okugawa S., Okamura N., Moriya K. (2013). Molecular characterization of carbapenemase-producing clinical isolates of Enterobacteriaceae in a teaching hospital, Japan. J. Med. Microbiol..

[177] Jousset A.B., Oueslati S., Bernabeu S., Takissian J., Creton E., Vogel A., Sauvadet A., Cotellon G., Gauthier L., Bonnin R.A. (2019). False-Positive Carbapenem-Hydrolyzing Confirmatory Tests Due to ACT-28, a Chromosomally Encoded AmpC with Weak Carbapenemase Activity from *Enterobacter kobei*. Antimicrob. Agents Chemother..

[178] Kim J.Y., Jung H.I., An Y.J., Lee J.H., Kim S.J., Jeong S.H., Lee K.J., Suh P.-G., Lee H.-S., Lee S.H. (2006). Structural basis for the extended substrate spectrum of CMY-10, a plasmid-encoded class C beta-lactamase. Mol. Microbiol..

[179] Jeon J.H., Hong M.K., Lee J.H., Lee J.J., Park K.S., Karim A.M., Jo J.Y., Kim J.H., Ko K.S., Kang L.W. (2014). Structure of ADC-68, a novel carbapenem-hydrolyzing class C extended-spectrum β-lactamase isolated from *Acinetobacter baumannii*. Acta Crystallogr. D Biol. Crystallogr..

[180] Rood I.G.H., Li Q. (2017). Review: Molecular detection of extended spectrum-β-lactamase- and carbapenemase-producing Enterobacteriaceae in a clinical setting. Diagn. Microbiol. Infect. Dis..

[181] Livermore D.M., Woodford N. (2006). The beta-lactamase threat in Enterobacteriaceae, *Pseudomonas* and *Acinetobacter*. Trends Microbiol..

[182] Durante-Mangoni E., Andini R., Zampino R. (2019). Management of carbapenem-resistant Enterobacteriaceae infections. Clin. Microbiol. Infect..

[183] Moxon C.A., Paulus S. (2016). Beta-lactamases in Enterobacteriaceae infections in children. J. Infect..

[184] Zafer M.M., El-Mahallawy H.A., Abdulhak A., Amin M.A., Al-Agamy M.H., Radwan H.H. (2019). Emergence of colistin resistance in multidrug-resistant *Klebsiella pneumoniae* and *Escherichia coli* strains isolated from cancer patients. Ann. Clin. Microbiol. Antimicrob..

[185] Ni W., Han Y., Liu J., Wei C., Zhao J., Cui J., Wang R., Liu Y. (2016). Tigecycline Treatment for Carbapenem-Resistant Enterobacteriaceae Infections: A Systematic Review and Meta-Analysis. Medicine.

[186] Sheu C.-C., Chang Y.-T., Lin S.-Y., Chen Y.-H., Hsueh P.-R. (2019). Infections Caused by Carbapenem-Resistant Enterobacteriaceae: An Update on Therapeutic Options. Front. Microbiol..

[187] Nordmann P. (2014). Carbapenemase-producing Enterobacteriaceae: Overview of a major public health challenge. Med. Mal. Infect..

[188] Suay-García B., Pérez-Gracia M.T. (2019). Present and Future of Carbapenem-resistant Enterobacteriaceae (CRE) Infections. Antibiotics.

[189] Rosa J.F., Rizek C., Marchi A.P., Guimaraes T., Miranda L., Carrilho C., Levin A.S., Costa S.F. (2017). Clonality, outer-membrane proteins profile and efflux pump in KPC- producing *Enterobacter* sp. in Brazil. BMC Microbiol..

[190] Pal A., Dhara L., Tripathi A. (2019). Contribution of acrB upregulation & OmpC/Ompk36 loss over the presence of blaNDM towards carbapenem resistance development among pathogenic *Escherichia coli* & *Klebsiella* spp.. Indian J. Med. Res..

[191] Chiu S.-K., Chan M.-C., Huang L.-Y., Lin Y.-T., Lin J.-C., Lu P.-L., Siu L.K., Chang F.-Y., Yeh K.-M. (2017). Tigecycline resistance among carbapenem-resistant *Klebsiella pneumoniae*: Clinical characteristics and expression levels of efflux pump genes. PLoS ONE.

[192] Ma P., Laibinis H.H., Ernst C.M., Hung D.T. (2018). Carbapenem Resistance Caused by High-Level Expression of OXA-663 β-Lactamase in an OmpK36-Deficient *Klebsiella pneumoniae* Clinical Isolate. Antimicrob. Agents Chemother..

[193] Hamzaoui Z., Ocampo-Sosa A., Fernandez Martinez M., Landolsi S., Ferjani S., Maamar E., Saidani M., Slim A., Martinez-Martinez L., Boutiba-Ben Boubaker I. (2018). Role of association of OmpK35 and OmpK36 alteration and blaESBL and/or blaAmpC genes in conferring carbapenem resistance among non-carbapenemase-producing *Klebsiella pneumoniae*. Int. J. Antimicrob. Agents.

[194] Chang Y.-T., Siu L.K., Wang J.-T., Wu T.-L., Chen Y.-H., Chuang Y.-C., Lin J.-C., Lu P.-L. (2019). Resistance mechanisms and molecular epidemiology of carbapenem-nonsusceptible *Escherichia coli* in Taiwan, 2012–2015. Infect. Drug Resist..

[195] Cantón R., Akóva M., Carmeli Y., Giske C.G., Glupczynski Y., Gniadkowski M., Livermore D.M., Miriagou V., Naas T., Rossolini G.M. (2012). Rapid evolution and spread of carbapenemases among Enterobacteriaceae in Europe. Clin. Microbiol. Infect..

[196] Guo X., Rao Y., Guo L., Xu H., Lv T., Yu X., Chen Y., Liu N., Han H., Zheng B. (2019). Detection and Genomic Characterization of a *Morganella morganii* Isolate from China That Produces NDM-5. Front. Microbiol..

[197] Moura Q., Cerdeira L., Fernandes M.R., Vianello M.A., Lincopan N. (2018). Novel class 1 integron (In1390) harboring blaGES-5 in a *Morganella morganii* strain recovered from a remote community. Diagn. Microbiol. Infect. Dis..

[198] Albiger B., Glasner C., Struelens M.J., Grundmann H., Monnet D.L. (2015). European Survey of Carbapenemase-Producing Enterobacteriaceae (EuSCAPE) working group Carbapenemase-producing Enterobacteriaceae in Europe: Assessment by national experts from 38 countries, May 2015. Eurosurveillance.

[199] Chen L., Mathema B., Chavda K.D., DeLeo F.R., Bonomo R.A., Kreiswirth B.N. (2014). Carbapenemase-producing *Klebsiella pneumoniae*: Molecular and genetic decoding. Trends Microbiol..

[200] Pitout J.D.D., Nordmann P., Poirel L. (2015). Carbapenemase-Producing *Klebsiella pneumoniae*, a Key Pathogen Set for Global Nosocomial Dominance. Antimicrob. Agents Chemother..

[201] Mathers A.J., Peirano G., Pitout J.D.D. (2015). The role of epidemic resistance plasmids and international high-risk clones in the spread of multidrug-resistant Enterobacteriaceae. Clin. Microbiol. Rev..

[202] Baquero F., Tedim A.P., Coque T.M. (2013). Antibiotic resistance shaping multi-level population biology of bacteria. Front. Microbiol..

[203] Woodford N., Turton J.F., Livermore D.M. (2011). Multiresistant Gram-negative bacteria: The role of high-risk clones in the dissemination of antibiotic resistance. FEMS Microbiol. Rev..

[204] Kitchel B., Rasheed J.K., Patel J.B., Srinivasan A., Navon-Venezia S., Carmeli Y., Brolund A., Giske C.G. (2009). Molecular epidemiology of KPC-producing *Klebsiella pneumoniae* isolates in the United States: Clonal expansion of multilocus sequence type 258. Antimicrob. Agents Chemother..

[205] Garza-Ramos U., Barrios H., Reyna-Flores F., Sánchez-Pérez A., Tamayo-Legorreta E., Ibarra-Pacheco A., Salazar-Salinas J., Núñez-Ceballos R., Silva-Sanchez J. (2014). Characteristics of KPC-2-producing *Klebsiella pneumoniae* (ST258) clinical isolates from outbreaks in 2 Mexican medical centers. Diagn. Microbiol. Infect. Dis..

[206] Tijet N., Sheth P.M., Lastovetska O., Chung C., Patel S.N., Melano R.G. (2014). Molecular characterization of *Klebsiella pneumoniae* carbapenemase (KPC)-producing Enterobacteriaceae in Ontario, Canada, 2008–2011. PLoS ONE.

[207] Andrade L.N., Curiao T., Ferreira J.C., Longo J.M., Clímaco E.C., Martinez R., Bellissimo-Rodrigues F., Basile-Filho A., Evaristo M.A., Del Peloso P.F. (2011). Dissemination of blaKPC-2 by the spread of *Klebsiella pneumoniae* clonal complex 258 clones (ST258, ST11, ST437) and plasmids (IncFII, IncN, IncL/M) among Enterobacteriaceae species in Brazil. Antimicrob. Agents Chemother..

[208] Prado-Vivar M.B., Ortiz L., Reyes J., Villacis E., Fornasini M., Baldeon M.E., Cardenas P.A. (2019). Molecular typing of a large nosocomial outbreak of KPC-producing bacteria in the biggest tertiary-care hospital of Quito, Ecuador. J. Glob. Antimicrob. Resist..

[209] Soria-Segarra C., González-Bustos P., López-Cerero L., Fernández-Cuenca F., Rojo-Martín M.D., Fernández-Sierra M.A., Gutiérrez-Fernández J. (2020). Tracking KPC-3-producing ST-258 *Klebsiella pneumoniae* outbreak in a third-level hospital in Granada (Andalusia, Spain) by risk factors and molecular characteristics. Mol. Biol. Rep..

[210] Meletis G., Chatzopoulou F., Fragkouli A., Alexandridou M., Mavrovouniotis I., Chatzinikolaou A., Chatzidimitriou D. (2019). Whole-genome sequencing study of KPC-encoding *Klebsiella pneumoniae* isolated in Greek private laboratories from non-hospitalised patients. J. Glob. Antimicrob. Resist..

[211] Becker L., Kaase M., Pfeifer Y., Fuchs S., Reuss A., Von Laer A., Sin M.A., Korte-Berwanger M., Gatermann S., Werner G. (2018). Genome-based analysis of Carbapenemase-producing *Klebsiella pneumoniae* isolates from German hospital patients, 2008–2014. Antimicrob. Resist. Infect. Control.

[212] Clemente A.M., Castronovo G., Antonelli A., D’Andrea M.M., Tanturli M., Perissi E., Paccosi S., Parenti A., Cozzolino F., Rossolini G.M. (2017). Differential Th17 response induced by the two clades of the pandemic ST258 *Klebsiella pneumoniae* clonal lineages producing KPC-type carbapenemase. PLoS ONE.

[213] Samuelsen Ø., Naseer U., Tofteland S., Skutlaberg D.H., Onken A., Hjetland R., Sundsfjord A., Giske C.G. (2009). Emergence of clonally related *Klebsiella pneumoniae* isolates of sequence type 258 producing plasmid-mediated KPC carbapenemase in Norway and Sweden. J. Antimicrob. Chemother..

[214] Yu F., Lv J., Niu S., Du H., Tang Y.-W., Pitout J.D.D., Bonomo R.A., Kreiswirth B.N., Chen L. (2018). Multiplex PCR Analysis for Rapid Detection of *Klebsiella pneumoniae* Carbapenem-Resistant (Sequence Type 258 [ST258] and ST11) and Hypervirulent (ST23, ST65, ST86, and ST375) Strains. J. Clin. Microbiol..

[215] Lee Y., Kim B.-S., Chun J., Yong J.H., Lee Y.S., Yoo J.S., Yong D., Hong S.G., D’Souza R., Thomson K.S. (2014). Clonality and Resistome analysis of KPC-producing *Klebsiella pneumoniae* strain isolated in Korea using whole genome sequencing. BioMed Res. Int..

[216] Jelić M., Hrenović J., Dekić S., Goić-Barišić I., Tambić Andrašević A. (2019). First evidence of KPC-producing ST258 *Klebsiella pneumoniae* in river water. J. Hosp. Infect..

[217] Leavitt A., Chmelnitsky I., Carmeli Y., Navon-Venezia S. (2010). Complete nucleotide sequence of KPC-3-encoding plasmid pKpQIL in the epidemic *Klebsiella pneumoniae* sequence type 258. Antimicrob. Agents Chemother..

[218] Chen L., Chavda K.D., Melano R.G., Jacobs M.R., Koll B., Hong T., Rojtman A.D., Levi M.H., Bonomo R.A., Kreiswirth B.N. (2014). Comparative genomic analysis of KPC-encoding pKpQIL-like plasmids and their distribution in New Jersey and New York Hospitals. Antimicrob. Agents Chemother..

[219] El Zowalaty M.E., Al Thani A.A., Webster T.J., El Zowalaty A.E., Schweizer H.P., Nasrallah G.K., Marei H.E., Ashour H.M. (2015). *Pseudomonas aeruginosa*: Arsenal of resistance mechanisms, decades of changing resistance profiles, and future antimicrobial therapies. Future Microbiol..

[220] Tümmler B. (2019). Emerging therapies against infections with *Pseudomonas aeruginosa*. F1000Research.

[221] Walters M.S., Grass J.E., Bulens S.N., Hancock E.B., Phipps E.C., Muleta D., Mounsey J., Kainer M.A., Concannon C., Dumyati G. (2019). Carbapenem-Resistant *Pseudomonas aeruginosa* at US Emerging Infections Program Sites, 2015. Emerg. Infect. Dis..

[222] Hong D.J., Bae I.K., Jang I.-H., Jeong S.H., Kang H.-K., Lee K. (2015). Epidemiology and Characteristics of Metallo-β-Lactamase-Producing *Pseudomonas aeruginosa*. Infect. Chemother..

[223] Eichenberger E.M., Thaden J.T. (2019). Epidemiology and Mechanisms of Resistance of Extensively Drug Resistant Gram-Negative Bacteria. Antibiot. Basel Switz..

[224] Potron A., Poirel L., Nordmann P. (2015). Emerging broad-spectrum resistance in *Pseudomonas aeruginosa* and *Acinetobacter baumannii*: Mechanisms and epidemiology. Int. J. Antimicrob. Agents.

[225] Hong J.S., Choi N., Kim S.J., Choi K.H., Roh K.H., Lee S. (2019). Molecular Characteristics of GES-Type Carbapenemase-Producing *Pseudomonas aeruginosa* Clinical Isolates from Long-Term Care Facilities and General Hospitals in South Korea. Microb. Drug Resist. Larchmt. N.

[226] McCracken M.G., Adam H.J., Blondeau J.M., Walkty A.J., Karlowsky J.A., Hoban D.J., Zhanel G.G., Mulvey M.R. (2019). Canadian Antimicrobial Resistance Alliance (CARA) and CANWARD Characterization of carbapenem-resistant and XDR *Pseudomonas aeruginosa* in Canada: Results of the CANWARD 2007–2016 study. J. Antimicrob. Chemother..

[227] Saharman Y.R., Pelegrin A.C., Karuniawati A., Sedono R., Aditianingsih D., Goessens W.H.F., Klaassen C.H.W., Van Belkum A., Mirande C., Verbrugh H.A. (2019). Epidemiology and characterisation of carbapenem-non-susceptible *Pseudomonas aeruginosa* in a large intensive care unit in Jakarta, Indonesia. Int. J. Antimicrob. Agents.

[228] Hishinuma T., Tada T., Kuwahara-Arai K., Yamamoto N., Shimojima M., Kirikae T. (2018). Spread of GES-5 carbapenemase-producing *Pseudomonas aeruginosa* clinical isolates in Japan due to clonal expansion of ST235. PLoS ONE.

[229] Bebrone C., Bogaerts P., Delbrück H., Bennink S., Kupper M.B., Rezende de Castro R., Glupczynski Y., Hoffmann K.M. (2013). GES-18, a new carbapenem-hydrolyzing GES-Type β-lactamase from *Pseudomonas aeruginosa* that contains Ile80 and Ser170 residues. Antimicrob. Agents Chemother..

[230] Hagemann J.B., Pfennigwerth N., Gatermann S.G., Von Baum H., Essig A. (2018). KPC-2 carbapenemase-producing *Pseudomonas aeruginosa* reaching Germany. J. Antimicrob. Chemother..

[231] De Oliveira Santos I.C., Albano R.M., Asensi M.D., D’Alincourt Carvalho-Assef A.P. (2018). Draft genome sequence of KPC-2-producing *Pseudomonas aeruginosa* recovered from a bloodstream infection sample in Brazil. J. Glob. Antimicrob. Resist..

[232] Shi L., Liang Q., Feng J., Zhan Z., Zhao Y., Yang W., Yang H., Chen Y., Huang M., Tong Y. (2018). Coexistence of two novel resistance plasmids, blaKPC-2-carrying p14057A and tetA(A)-carrying p14057B, in *Pseudomonas aeruginosa*. Virulence.

[233] Wolter D.J., Khalaf N., Robledo I.E., Vázquez G.J., Santé M.I., Aquino E.E., Goering R.V., Hanson N.D. (2009). Surveillance of carbapenem-resistant *Pseudomonas aeruginosa* isolates from Puerto Rican Medical Center Hospitals: Dissemination of KPC and IMP-18 beta-lactamases. Antimicrob. Agents Chemother..

[234] Jovcic B., Lepsanovic Z., Suljagic V., Rackov G., Begovic J., Topisirovic L., Kojic M. (2011). Emergence of NDM-1 metallo-β-lactamase in *Pseudomonas aeruginosa* clinical isolates from Serbia. Antimicrob. Agents Chemother..

[235] Flateau C., Janvier F., Delacour H., Males S., Ficko C., Andriamanantena D., Jeannot K., Merens A., Rapp C. (2012). Recurrent pyelonephritis due to NDM-1 metallo-beta-lactamase producing *Pseudomonas aeruginosa* in a patient returning from Serbia, France, 2012. Eurosurveillance.

[236] Ismail S.J., Mahmoud S.S. (2018). First detection of New Delhi metallo-β-lactamases variants (NDM-1, NDM-2) among *Pseudomonas aeruginosa* isolated from Iraqi hospitals. Iran. J. Microbiol..

[237] Urbanowicz P., Izdebski R., Baraniak A., Żabicka D., Ziółkowski G., Hryniewicz W., Gniadkowski M. (2019). *Pseudomonas aeruginosa* with NDM-1, DIM-1 and PME-1 β-lactamases, and RmtD3 16S rRNA methylase, encoded by new genomic islands. J. Antimicrob. Chemother..

[238] Chew K.L., Octavia S., Ng O.T., Marimuthu K., Venkatachalam I., Cheng B., Lin R.T.P., Teo J.W.P. (2019). Challenge of drug resistance in *Pseudomonas aeruginosa*: Clonal spread of NDM-1-positive ST-308 within a tertiary hospital. J. Antimicrob. Chemother..

[239] Liew S.M., Rajasekaram G., Puthucheary S.D., Chua K.H. (2018). Detection of VIM-2-, IMP-1- and NDM-1-producing multidrug-resistant *Pseudomonas aeruginosa* in Malaysia. J. Glob. Antimicrob. Resist..

[240] Pollini S., Maradei S., Pecile P., Olivo G., Luzzaro F., Docquier J.-D., Rossolini G.M. (2013). FIM-1, a new acquired metallo-β-lactamase from a *Pseudomonas aeruginosa* clinical isolate from Italy. Antimicrob. Agents Chemother..

[241] Boyd D.A., Lisboa L.F., Rennie R., Zhanel G.G., Dingle T.C., Mulvey M.R. (2019). Identification of a novel metallo-β-lactamase, CAM-1, in clinical *Pseudomonas aeruginosa* isolates from Canada. J. Antimicrob. Chemother..

[242] Sevillano E., Gallego L., García-Lobo J.M. (2009). First detection of the OXA-40 carbapenemase in P. aeruginosa isolates, located on a plasmid also found in A. baumannii. Pathol. Biol..

[243] El Garch F., Bogaerts P., Bebrone C., Galleni M., Glupczynski Y. (2011). OXA-198, an acquired carbapenem-hydrolyzing class D beta-lactamase from *Pseudomonas aeruginosa*. Antimicrob. Agents Chemother..

[244] Esenkaya Taşbent F., Özdemir M. (2015). The presence of OXA type carbapenemases in Pseudomonas strains: First report from Turkey. Mikrobiyol. Bul..

[245] Rouhi S., Ramazanzadeh R. (2018). Prevalence of blaOxacillinase-23 and blaOxacillinase-24/40-type Carbapenemases in *Pseudomonas aeruginosa* Species Isolated from Patients with Nosocomial and Non-nosocomial Infections in the West of Iran. Iran. J. Pathol..

[246] Doi Y., Murray G.L., Peleg A.Y. (2015). *Acinetobacter baumannii*: Evolution of antimicrobial resistance-treatment options. Semin. Respir. Crit. Care Med..

[247] Simo Tchuinte P.L., Rabenandrasana M.A.N., Kowalewicz C., Andrianoelina V.H., Rakotondrasoa A., Andrianirina Z.Z., Enouf V., Ratsima E.H., Randrianirina F., Collard J.-M. (2019). Phenotypic and molecular characterisations of carbapenem-resistant *Acinetobacter baumannii* strains isolated in Madagascar. Antimicrob. Resist. Infect. Control.

[248] Nowak P., Paluchowska P. (2016). *Acinetobacter baumannii*: Biology and drug resistance—Role of carbapenemases. Folia Histochem. Cytobiol..

[249] Karthikeyan K., Thirunarayan M.A., Krishnan P. (2010). Coexistence of blaOXA-23 with blaNDM-1 and armA in clinical isolates of *Acinetobacter baumannii* from India. J. Antimicrob. Chemother..

[250] Chen Y., Zhou Z., Jiang Y., Yu Y. (2011). Emergence of NDM-1-producing *Acinetobacter baumannii* in China. J. Antimicrob. Chemother..

[251] Decousser J.W., Jansen C., Nordmann P., Emirian A., Bonnin R.A., Anais L., Merle J.C., Poirel L. (2013). Outbreak of NDM-1-producing *Acinetobacter baumannii* in France, January to May 2013. Eurosurveillance.

[252] Voulgari E., Politi L., Pitiriga V., Dendrinos J., Poulou A., Georgiadis G., Tsakris A. (2016). First report of an NDM-1 metallo-β-lactamase-producing *Acinetobacter baumannii* clinical isolate in Greece. Int. J. Antimicrob. Agents.

[253] Hammerum A.M., Larsen A.R., Hansen F., Justesen U.S., Friis-Møller A., Lemming L.E., Fuursted K., Littauer P., Schønning K., Gahrn-Hansen B. (2012). Patients transferred from Libya to Denmark carried OXA-48-producing *Klebsiella pneumoniae*, NDM-1-producing *Acinetobacter baumannii* and meticillin-resistant Staphylococcus aureus. Int. J. Antimicrob. Agents.

[254] Bogaerts P., Rezende de Castro R., Roisin S., Deplano A., Huang T.-D., Hallin M., Denis O., Glupczynski Y. (2012). Emergence of NDM-1-producing *Acinetobacter baumannii* in Belgium. J. Antimicrob. Chemother..

[255] Krizova L., Bonnin R.A., Nordmann P., Nemec A., Poirel L. (2012). Characterization of a multidrug-resistant *Acinetobacter baumannii* strain carrying the blaNDM-1 and blaOXA-23 carbapenemase genes from the Czech Republic. J. Antimicrob. Chemother..

[256] Bonnin R.A., Poirel L., Naas T., Pirs M., Seme K., Schrenzel J., Nordmann P. (2012). Dissemination of New Delhi metallo-β-lactamase-1-producing *Acinetobacter baumannii* in Europe. Clin. Microbiol. Infect..

[257] Ghazawi A., Sonnevend A., Bonnin R.A., Poirel L., Nordmann P., Hashmey R., Rizvi T.A., B Hamadeh M., Pál T. (2012). NDM-2 carbapenemase-producing *Acinetobacter baumannii* in the United Arab Emirates. Clin. Microbiol. Infect..

[258] Park Y.K., Jung S.-I., Park K.-H., Kim S.H., Ko K.S. (2012). Characteristics of carbapenem-resistant Acinetobacter spp. other than *Acinetobacter baumannii* in South Korea. Int. J. Antimicrob. Agents.

[259] Mugnier P.D., Poirel L., Nordmann P. (2009). Functional analysis of insertion sequence ISAba1, responsible for genomic plasticity of *Acinetobacter baumannii*. J. Bacteriol..

[260] Zhao Y., Hu K., Zhang J., Guo Y., Fan X., Wang Y., Mensah S.D., Zhang X. (2019). Outbreak of carbapenem-resistant *Acinetobacter baumannii* carrying the carbapenemase OXA-23 in ICU of the eastern Heilongjiang Province, China. BMC Infect. Dis..

[261] Caldart R.V., Fonseca E.L., Freitas F., Rocha L., Vicente A.C. (2019). *Acinetobacter baumannii* infections in Amazon Region driven by extensively drug resistant international clones, 2016–2018. Mem. Inst. Oswaldo Cruz.

[262] Ewers C., Klotz P., Leidner U., Stamm I., Prenger-Berninghoff E., Göttig S., Semmler T., Scheufen S. (2017). OXA-23 and ISAba1-OXA-66 class D β-lactamases in *Acinetobacter baumannii* isolates from companion animals. Int. J. Antimicrob. Agents.

[263] Al-Agamy M.H., Jeannot K., El-Mahdy T.S., Shibl A.M., Kattan W., Plésiat P., Courvalin P. (2017). First Detection of GES-5 Carbapenemase-Producing *Acinetobacter baumannii* Isolate. Microb. Drug Resist. Larchmt. N.

[264] Bonnin R.A., Nordmann P., Potron A., Lecuyer H., Zahar J.-R., Poirel L. (2011). Carbapenem-hydrolyzing GES-type extended-spectrum beta-lactamase in *Acinetobacter baumannii*. Antimicrob. Agents Chemother..

[265] Bonnin R.A., Rotimi V.O., Al Hubail M., Gasiorowski E., Al Sweih N., Nordmann P., Poirel L. (2013). Wide dissemination of GES-type carbapenemases in *Acinetobacter baumannii* isolates in Kuwait. Antimicrob. Agents Chemother..

[266] Robledo I.E., Aquino E.E., Santé M.I., Santana J.L., Otero D.M., León C.F., Vázquez G.J. (2010). Detection of KPC in *Acinetobacter* spp. in Puerto Rico. Antimicrob. Agents Chemother..

[267] Martínez T., Vázquez G.J., Aquino E.E., Martínez I., Robledo I.E. (2014). ISEcp1-mediated transposition of blaKPC into the chromosome of a clinical isolate of *Acinetobacter baumannii* from Puerto Rico. J. Med. Microbiol..

[268] Périchon B., Goussard S., Walewski V., Krizova L., Cerqueira G., Murphy C., Feldgarden M., Wortman J., Clermont D., Nemec A. (2014). Identification of 50 class D β-lactamases and 65 Acinetobacter-derived cephalosporinases in *Acinetobacter* spp.. Antimicrob. Agents Chemother..

[269] Papp-Wallace K.M., Becka S.A., Zeiser E.T., Ohuchi N., Mojica M.F., Gatta J.A., Falleni M., Tosi D., Borghi E., Winkler M.L. (2017). Overcoming an Extremely Drug Resistant (XDR) Pathogen: Avibactam Restores Susceptibility to Ceftazidime for Burkholderia cepacia Complex Isolates from Cystic Fibrosis Patients. ACS Infect. Dis..

[270] Chi X., Zhang J., Xu H., Yu X., Shen P., Ji J., Ying C., Zheng B., Xiao Y. (2019). Emergence of KPC-2-producing Raoultella ornithinolytica isolated from hospital wastewater treatment plant. Antimicrob. Agents Chemother..

[271] Seng P., Boushab B.M., Romain F., Gouriet F., Bruder N., Martin C., Paganelli F., Bernit E., Le Treut Y.P., Thomas P. (2016). Emerging role of Raoultella ornithinolytica in human infections: A series of cases and review of the literature. Int. J. Infect. Dis..

[272] Kieffer N., Guzmán-Puche J., Poirel L., Kang H.J., Jeon C.O., Nordmann P. (2019). ZHO-1, an intrinsic MBL from the environmental Gram-negative species Zhongshania aliphaticivorans. J. Antimicrob. Chemother..

[273] Kieffer N., Poirel L., Fournier C., Haltli B., Kerr R., Nordmann P. (2019). Characterization of PAN-1, a Carbapenem-Hydrolyzing Class B β-Lactamase from the Environmental Gram-Negative Pseudobacteriovorax antillogorgiicola. Front. Microbiol..

